# Core–Shell Plasmonic Nanocomposites with Synergistic Photothermal and Photochemical Activity for Biomedical Applications

**DOI:** 10.3390/nano16030174

**Published:** 2026-01-27

**Authors:** Anca Roibu, Florina Silvia Iliescu, Ana-Maria Zamfirescu, Elena Radu, Laura-Elena Andrei, Amarachi Rosemary Osi, Georgeta-Luminița Gheorghiu, Cornel Cobianu, Ciprian Iliescu

**Affiliations:** 1 eBio-Hub Centre of Excellence in Bioengineering, National University of Science and Technology Politehnica Bucharest, 060042 Bucharest, Romania; anca.roibu@upb.ro (A.R.); florina.iliescu@upb.ro (F.S.I.); ana.zamfirescu0803@upb.ro (A.-M.Z.); elena.radu2211@upb.ro (E.R.); laura.andrei2507@upb.ro (L.-E.A.); amarachi.osi@upb.ro (A.R.O.); gheorghiu.luminita@gmail.com (G.-L.G.); 2 National Institute for Research and Development in Microtechnologies—IMT Bucharest, 077190 Voluntari, Romania; 3 Faculty of Material Science and Engineering, National University of Science and Technology Politehnica Bucharest, Splaiul Independentei 313, 060042 Bucharest, Romania; 4 Academy of Romanian Scientists, Ilfov 3, 050044 Bucharest, Romania

**Keywords:** core–shell nanostructures, nanocomposite, plasmonic effect, photothermal effect, photocatalytic effect, antimicrobial, cancer therapy, wound healing

## Abstract

Nanomedicine changes our lives by impacting diagnostics and therapeutics. In the biomedical domain, core–shell nanostructures have significant potential for photothermal therapy, diagnostics, sensing, drug delivery, and imaging. This work reviews the synergistic photothermal and photochemical effects of core–shell nanocomposites in the biomedical field. Several historical points in the development of nanostructures and fundamental core–shell plasmonic nanocomposites are provided in the introductory sections. Further, we analyzed the core–shell construction and its main biomedical applications: antimicrobial, cancer therapy, wound healing, and tissue regeneration. Moreover, we present relevant design considerations, performance optimization, and toxicity studies focused on synergistic photothermal–photochemical effects. Despite the promising biomedical research, several challenges remain before core–shell nanocomposites are widely translated into clinical settings and highlight the potential from technological and legal perspectives. The review concludes by outlining the pathways by which the synergistic photothermal–photochemical response of the core–shell nanocomposites plays a key role in nanomedicine and personalized medicine.

## 1. Introduction

Nanomedicine profoundly impacts healthcare by enabling early and more sensitive diagnosis, targeted and safer drug delivery, accelerating regenerative medicine, and creating new antimicrobial and vaccine strategies. Ultimately, working at the molecular level enables personalized medicine for diseases such as cancer, neurological disorders, and infections, thereby improving outcomes and quality of life [1].

Plasmonic nanomaterials are considered an exciting new class of functional platforms in nanomedicine due to their unique light–matter interactions and programmable physicochemical features [2]. Their biomedical applications began to grow steadily about two decades ago as an extension of scientific discoveries and well-established research in plasmonic optics and photocatalytic materials [3]. Metallic nanostructures (e.g., gold, silver, and alloys) exposed to resonant electromagnetic radiation exhibit localized surface plasmon resonance (LSPR) [4] for many biological applications, such as photothermal therapy [5], imaging [6], biosensing [7], and controlled drug release [8]. Table 1 presents the key milestones in the convergence of the photothermal and photocatalytic fields. Nevertheless, despite this significant progress, several challenges have plagued the practical implementation of single-component plasmonics, including low treatment efficacy, low photostability, and limited multifunctionality.

Core–shell nanocomposites hold great potential to overcome such limitations by integrating complementary materials into a hierarchical nanosystem [18]. By judiciously combining plasmonic cores with functional shell components (e.g., semiconductors, polymers, carbon-based materials, or metal–organic frameworks), hybrid structures that display synergistic photothermal and photochemical performance can be designed. In addition to retaining the strong plasmonic response of a metallic core, these structures could exploit the enhanced charge separation, hot-electron transfer, or catalytic activity of the shell material. Such synergistic effects may increase the generation of reactive oxygen species (ROS) for photodynamic therapy (PDT), improve light-triggered drug delivery, or enhance contrast in multimodal imaging [19]. Moreover, core–shell architectures allow fine-tuning of biocompatibility, colloidal stability, and biodistribution, which are critical for safe and effective in vivo function [20]. Recently, a combination of photothermal and photochemical modalities delivered via a single nanoplatform yielded superior therapeutic properties compared with monotherapy [21]. Significantly, while photothermal heating technology can disrupt tumor microenvironments and increase cellular susceptibility to oxidative damage, photochemical processes can reduce the thermal dose required for therapy, thereby reducing injury to surrounding healthy tissues. This aspect is particularly beneficial in biomedical settings where precise, minimally invasive and tunable therapeutic strategies are needed, with synergistic interplay being crucial. As a result, core–shell plasmonic nanocomposites have emerged as highly versatile platforms capable of integrating diagnosis, therapy, and controlled biological interaction. Their multifunctionality, combined with the ability to tailor optical properties across the visible to near-infrared (NIR) spectrum, positions them as promising candidates for next-generation photonic nanomedicine.

Here, we review the design principles and the mechanistic bases for core–shell plasmonic nanocomposites with synergistic photothermal and photochemical properties, and their application in biomedicine. Special focus is given to material engineering approaches, to synergistic mechanisms of action, and potential new fields in cancer therapy, antimicrobial treatment, and targeted theranostics. We further highlight the field’s key difficulties and perspectives. Our current review integrates core–shell architecture, plasmonic hot-carrier photothermal and photocatalytic/photodynamic processes, and biomedical performance into a unified framework, for multiple applications. This review fills that gap by systematically linking core–shell composition, geometry, and interfacial electronic structure to synergistic photothermal and photochemical outputs, and by correlating these mechanisms with outcomes in cancer therapy, antimicrobial activity, and wound healing. We further highlight the design rules, biological constraints, and translational considerations required to develop plasmonic core–shell nanocomposites capable of delivering robust, clinically relevant photothermal–photochemical synergy.

## 2. Fundamentals of Core–Shell Plasmonic Nanocomposites

### 2.1. Structure and Synthesis

Core–shell nanostructures are hybrid nanoparticles (NPs) composed of two or more components, with one component serving as the core and one or more layers forming the shell. This configuration allows combining different components to obtain enhanced or new properties that the individual materials cannot. Plasmonic core–shell NPs include at least one plasmonic material such as Au, Ag, Cu, and CuS in their structure. At the same time, the other components may consist of additional plasmonic metals, silica, semiconductors, or polymers. The synthesis of these hybrid NPs can be performed using Turkovich [22], seed-mediated growth [23,24], sol-gel [25], hydrothermal [26], pulse laser ablation in liquid (PLAL) [27,28], and self-assembly methods [29]. Two commonly used synthesis techniques are:

- the Turkevich method to produce colloidal gold NPs through reduction of chloroauric acid using trisodium citrate [30], and
- Stöber (sol-gel) method to synthesize SiO_2_ with controllable and uniform size using tetraethyl orthosilicate (TEOS), ammonia, and water [31].

Several reviews [32,33] previously addressed the synthesis of plasmonic core–shell NPs; therefore, it will not be further developed in this survey.

### 2.2. Mechanisms of LSPR-Induced Photothermal and Photochemical Effects

**Figure 1:**
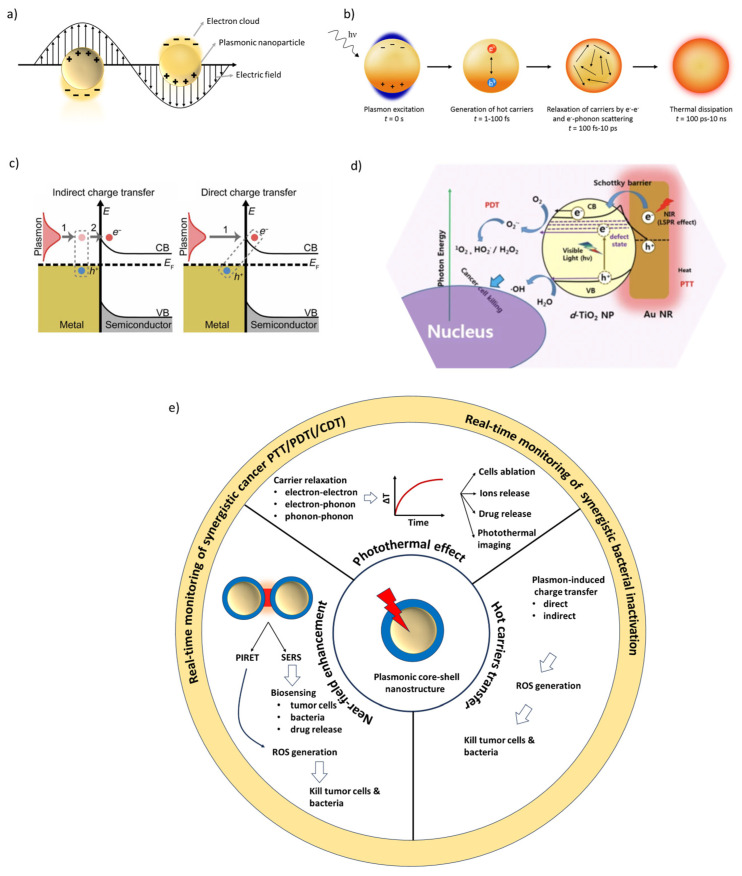
(**a**) The collective oscillation of free electrons around a plasmonic nanoparticle upon light irradiation. (**b**) Schematic representation of the plasmon excitation and decay. Adapted from [35]. (**c**) Schematic representation of indirect and direct plasmon-induced charge transfers. The numbers 1 and 2 indicate the number of sequential steps. Adapted from [54]. (**d**) The generation of ROS by simultaneous irradiation with visible and near-infrared light of Au nanorods/TiO_2_ to kill cancer cells. Adapted from [14]. (**e**) Summary of the key plasmon-induced mechanisms encountered in biomedical applications.

LSPR occurs when metallic NPs smaller than the wavelength of incident light are irradiated, and their conduction electrons coherently oscillate (Figure 1a). Resonance occurs when the frequency of the incident light matches the collective oscillation frequency of these electrons, leading to strong light absorption and scattering, as well as the generation of highly amplified, localized electric fields near the nanoparticle surface [34].

There are several mechanisms by which LSPR exerts its photothermal or photocatalytic effects. One of them (Figure 1b) involves charge carriers: after plasmon excitation and charge-carrier generation, they undergo electron–electron, electron–phonon, and phonon–phonon relaxation processes. Electron–phonon scattering causes heating of the nanoparticle, while phonon–phonon scattering leads to thermal dissipation into the surrounding medium [35]. The resulting photothermal effect has been extensively used in biomedical applications, including tumoral cell death (e.g., photothermal therapy), microbial inactivation, controlled ions and drug release, and photothermal imaging [36]. Another mechanism in plasmonic photocatalysis is hot charge carrier generation and transfer. LSPR decay generates hot electrons and holes that can be indirectly or directly injected into adsorbed molecules that could undergo redox reactions on the surface of the metal nanoparticles, or into semiconductors (in direct contact) to drive charge-transfer processes (Figure 1c) [37]. The separated electrons and holes can react with the molecular O_2_ and H_2_O to produce ROS, such as hydroxyl radicals (^·^OH), singlet oxygen (^1^O_2_), hydrogen peroxide (H_2_O_2_), and superoxide radical $\left(O_{2}^{\cdot -}\right)$. The generated ROS can kill bacteria and malignant cells (Figure 1d) [14]. A third mechanism in plasmonic photocatalysis, near-field enhancement, occurs when strong electromagnetic fields generated around metallic NPs during surface plasmon resonance enhance the local electromagnetic field intensity. When it arises between two neighboring NPs, it is commonly referred to as “hot-spots”. For instance, Plasmon-Induced Resonance Energy Transfer (PIRET) is an energy transfer process that occurs between the plasmonic metal NP and an acceptor located in the near-field, such as a semiconductor or a photosensitizer, when their absorption band overlaps with that of the plasmonic metal [38]. PIRET mechanism facilitates the formation of ROS that can be utilized in cancer and antimicrobial treatments [39]. In addition, surface-enhanced Raman scattering (SERS) utilizes near-field enhancement to amplify the inherently weak Raman signal of molecules by up to several orders of magnitude, enabling the detection of cancer cells, bacteria, and drugs with high sensitivity [40]. Figure 1e summarizes the plasmonic-induced mechanisms for core–shell NPs in biomedical applications.

Importantly, several synergies could emerge when two or more mechanisms are coupled:

- Photothermal (PTT) cancer cells ablation and ROS generated through PIRET/hot-carriers transfer lead to synergistic PTT/PDT cancer therapy [41].
- Photothermal cells ablation and heat-induced drug-release lead to synergistic PTT/(Chemodynamic therapy (CDT)) of cancer [42,43].
- Photothermal cell ablation and heat-induced ion release lead to synergistic bacterial inactivation [44].

Moreover, photothermal or photochemical routes were often coupled with LSPR sensing for real-time monitoring during cancer therapies [45] and bacterial inactivation [46].

To identify and quantify the underlying mechanisms, several approaches are commonly employed:

- Radical scavengers such as sodium azide (NaN_3_) to investigate the formation of ROS [47].
- ROS detection dyes, including carboxy-H2DFFDA [48] and Singlet Oxygen Sensor Green (SOSG) probes to detect singlet oxygen (despite reported specificity concerns) [49].
- Selective reactions such as the photooxidation of ABDA, a singlet oxygen-specific probe [50].
- Temperature measurement under irradiation, using thermal sensors [49] or infrared imaging devices [51,52].
- Carefully designed in vitro and in vivo control experiments, where formulations are adjusted so that only photothermal or photochemical pathways are activated [53].

Despite the availability of these methods, most studies involving plasmonic nanoparticles for biomedical applications report only some of them. This makes it challenging to conclude about the involved mechanisms —particularly in cancer therapies, where biological environments are complex and multifunctional nanostructures are involved. More comprehensive mechanistic studies that map light-induced effects would greatly support the development of advanced plasmonic materials and more effective therapeutic strategies.

### 2.3. Tuning LSPR in Core–Shell Nanostructures

**Figure 2:**
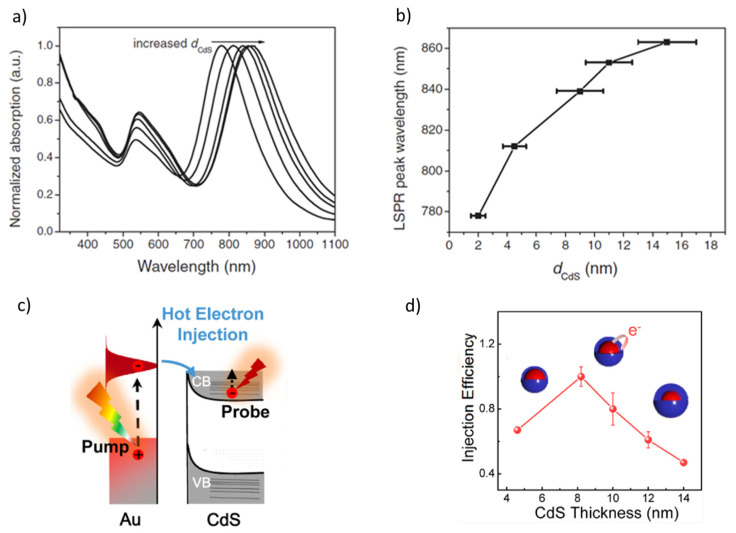
Optical properties for different plasmonic core–shell nanostructure sizes. (**a**) Normalized absorption spectra of Au@CdS core–shell nanorods as a function of CdS shell diameter (d_CdS_), (**b**) LSPR peak wavelength of Au@CdS versus d_CdS_. a and b reproduced with permission from [59], Copyright 2011 Wiley-VCH. (**c**) Monitoring the hot-electron injection process for Au@CdS after ultrafast photoexcitation of the Au LSPR band, (**d**) Hot-electron injection efficiency versus shell thickness for Au@CdS. (**c**,**d**) Reproduced with permission from [61], Copyright 2021 American Chemical Society.

The plasmonic properties of core–shell NPs with a given composition can be tailored by controlling the size [55], morphology [56,57], and the nature of the core–shell interface [58]. Of these approaches, adjusting size and shape was primarily used to achieve the desired performance in biomedical applications. Generally, increasing the shell thickness and plasmonic core size leads to a redshift of the LSPR absorbance band (Figure 2a,b) [59,60]. For Au@CdS, the hot-electron injection efficiency firstly increased with the thickness of a semiconductor shell, but it decreased after a critical point (Figure 2c,d) [61]. Similarly, the optimal 4 nm TiO_2_ shell thickness of Au@TiO_2_ NPs [62] led to the highest SERS enhancement and photocatalytic activity, explained by the near-field enhancement induced by the plasmonic coupling between neighboring particles. Moreover, when the plasmonic metal was placed on the shell, as in SiO_2_@Au, the LSPR band redshifted by several hundreds of nanometers by varying the relative dimensions of the SiO_2_ core and Au shell. Through this method, the absorption in the infrared domain that is essential for cancer therapies becomes easily achieved [63].

**Figure 3:**
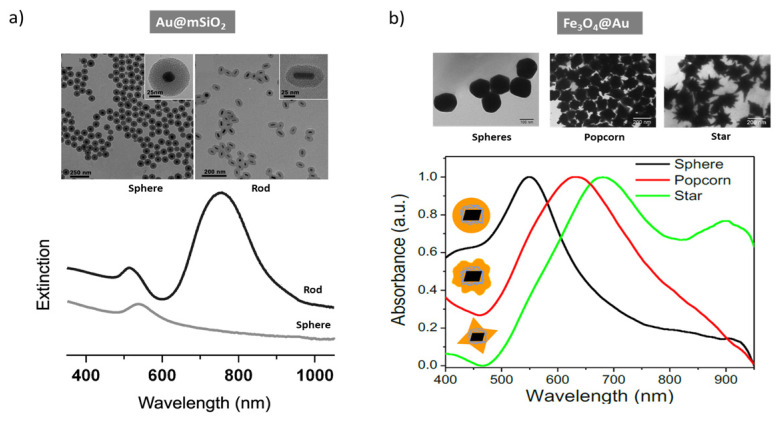
Optical properties for different plasmonic core–shell nanostructure morphologies. (**a**) TEM images and optical properties of core–shell Au@mSiO_2_ with the shapes of sphere and rod. Adapted with permission from [56], Copyright 2016 Royal Society of Chemistry. (**b**) TEM images and optical properties of core–shell Fe_3_O_4_@Au with the shapes of sphere, popcorn, and star. Adapted with permission from [23], Copyright 2016 American Chemical Society.

Furthermore, controlling the shape of the core and shell enabled accessing the infrared region (Figure 3). For Au@mesoporous SiO_2_, the spherical Au core leads to an LSPR absorption band in the visible range [56]. On the other hand, the Au nanorod core supported two LSPR bands in the visible (transverse plasmon peak) and infrared (longitudinal plasmon peak) regions, tunable by adjusting the rod’s diameter-to-length ratio. The strong NIR absorption of Au nanorod-based composites makes them useful in applications that require deep light penetration. However, the more than 1-day synthesis of rod-like core–shell nanoparticles, compared with only 2 h for sphere production, made core–shell spheres preferable for scale-up [56]. The plasmonic properties of different shapes, such as spheres, popcorns, and stars, were compared using core−shell Fe_3_O_4_@Au nanoparticles. As in the case of nanorods, the nanopopcorns and nanostars exhibited redshifted LSPR bands compared with the nanospheres, due to shape anisotropy [23].

## 3. Core–Shell Architectures in Biomedical Applications

### 3.1. Overview

**Figure 4:**
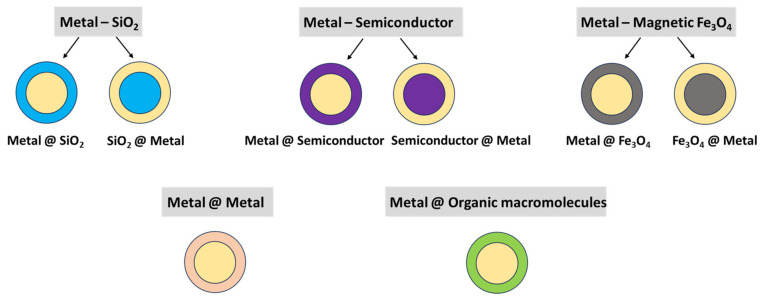
Schematic representation of plasmonic core–shell architectures used in biomedical applications. The yellow color indicates the location of the plasmonic metal.

The emerging transition from plasmonic NPs, like Au and Ag, to core–shell plasmonic nanocomposites was motivated by the need to further increase the performance, long-term stability, and biocompatibility of the plasmonic nanohybrids in different biomedical applications. The major plasmonic core–shell nanostructures used in biomed applications and their architectures are described below and shown in Table 2 and Figure 4.

### 3.2. Metal-SiO_2_

Since changes in temperature and reaction conditions alter the morphology and properties of plasmonic nanoparticles, leading to their loss of functionality [78,79], new designs were considered to stabilize the NPs. For instance, Xie et al. reported higher thermal stability for Au nanotriangles with mesoporous silica shells than for uncoated nanoparticles [79]. Dhanalekshmi et al. synthesized core–shell Ag@SiO_2_ nanoparticles, conducted PDT in the human cervical cancer cell line (HeLa) [47], and demonstrated that the process of photohemolysis was primarily dependent on the generation of the singlet oxygen, (^1^O_2_). When Macia et al. added Rose Bengal (RB) as a photosensitizer to Ag nanocubes @ SiO_2_, they found 12 times the amount of singlet oxygen than when RB was used alone and a stronger local enhanced electric field of Ag nanocubes compared with Ag nanospheres [50]. They evaluated the antimicrobial activity of Ag nanocubes@SiO_2_-RB against Gram-positive *S. aureus* and Gram-negative *E. coli* and found it to be higher than that of Ag nanosphere@SiO_2_-RB. Similarly, integrating photosensitizers, such as methylene blue [48], porphyrin [49,64], IR795 [51], and RB [50] with core–shell Au@SiO_2_ to enhance singlet oxygen production and obtain a PDT/PTT synergism was attempted. PTT was based on the photothermal energy conversion of the plasmonic metal nanocore. The increased production of ^1^O_2_ correlated well with a good energy overlap on the surface plasmon resonance (SPR) of the metal [48], high scattering yields [50], and an optimum distance between the metal and the photosensitizer [80]. An additional layer of Au coated on top of the core–shell Au@SiO_2_ enhanced the SPR signal, augmented the photothermal conversion into PTT, and improved sensing [81].

**Figure 5:**
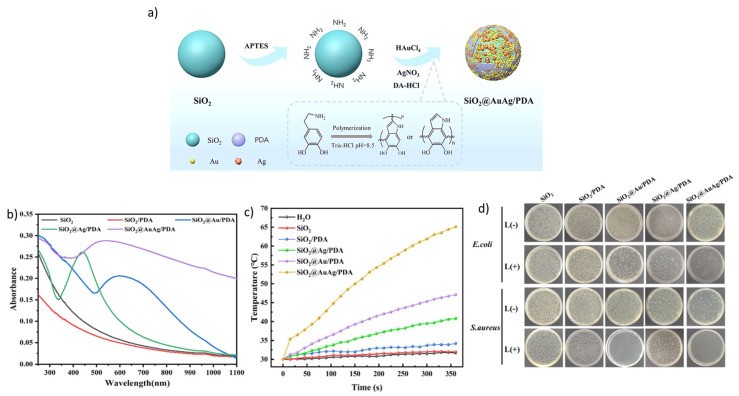
(**a**) Schematic illustration for the preparation of SiO_2_@AuAg/PDA core/shell nanospheres, (**b**) UV–Vis absorption spectra of SiO_2_, SiO_2_/PDA, SiO_2_@Au/PDA, SiO_2_@Ag/PDA, and SiO_2_@AuAg/PDA, (**c**) Temperature elevation curves for DI water, SiO_2_, SiO_2_/PDA, SiO_2_@Ag/PDA, SiO_2_@Au/PDA, and SiO_2_@AuAg/PDA under NIR laser irradiation (808 nm), (**d**) Images of *E. coli* and *S. aureus* colonies treated with SiO_2_, SiO_2_/PDA, SiO_2_@Au/PDA, SiO_2_@Ag/PDA, and SiO_2_@AuAg/PDA with or without NIR irradiation (808 nm) for 6 min. L(+) and L(−) indicate the presence and absence of light, respectively. Adapted from [44].

Meanwhile, SiO_2_@Au NPs, with a silica core and an Au-coated shell, frequently used in biomedical applications [29,82], permit the easy development of materials with the desired light absorption in the near-infrared (NIR) domain by tuning the core and shell sizes (see Section 2.2) [63,83]. With a 120 nm core diameter, an Au shell thickness of 12–15 nm, and polyethylene glycol (PEG) coated to improve blood circulation time, SiO_2_@Au NPs were biocompatible when injected into mice [84] and, when similarly sized, were investigated for photothermal performance in a prostate cancer clinical trial [85]. Moreover, an additional layer of SiO_2_ upon the SiO_2_@ Au nanostructure prevented Au nanoparticle loss due to physical stress and enhanced the photothermal effect on tumor volume [22]. The good photothermal effect of Au and Ag and the increased release of Ag^+^ by NIR irradiation were demonstrated by Ci et al. [44]. They reported core–shell nanospheres of SiO_2_@AuAg/polydopamine (PDA) that exhibited a synergistic photothermal–Ag^+^-release antibacterial effect in the NIR domain. The composite was prepared using a one-step polymerization of a hybrid AuAg/PDA layer over SiO_2_ nanospheres (Figure 5a). The SiO_2_@AuAg/PDA particles with a wide absorption in the UV–Vis-NIR region, and a characteristic peak absorption at around 550 nm (Figure 5c,d), exhibited the highest photothermal behavior that inactivated *E. coli* and *S. aureus* with illumination at 808 nm.

### 3.3. Metal-Semiconductor

Integrating plasmonic NPs with semiconductors in core–shell nanostructures improved stability under photothermal conditions, light harvesting, and charge transfer. So far, semiconductor-based core–shell composites of noble metals have demonstrated significantly enhanced photocatalytic activity in hydrogen generation, CO_2_ conversion, pollutant degradation, and gas and liquid analyte detection [86]. Core–shell materials can be prepared from a broad set of semiconductors, including TiO_2_ [87,88], g-C_3_N_4_ [89], ZnO [90], MnO_2_ [52], Cu_2_O [91], and CuS [92]. For instance, TiO_2_ and ZnO exhibited good biocompatibility and UV activity due to their wide band gaps (e.g., 3–3.2 eV and 3.37 eV, respectively) [93,94,95]. In the meantime, the presence of plasmonic NPs broadened the composite’s spectral response, from the visible to the NIR. Moreover, Au@TiO_2_ loaded with doxorubicin (DOX) showed synergistic PTT and PDT effects for cancer treatment at 635 nm. Even more interesting, Core–shell Au@ZnO nanostructures were employed for their antibiotic and cytostatic activities in the absence of light [41]. As a result, the addition of ZnO to plasmonic PTT and PDT will soon be the subject of several biomedical studies. Carbon nitride is another semiconductor that, as a PDT photosensitizer, can be coupled with plasmonic PTT components for synergistic potential in cancer therapy [96]. Other semiconductors could be used in cancer therapy when functionalized. For instance, Au@MnO_2_ NPs functionalized with a CCK peptide simultaneously achieved PTT and SERS to monitor MnO_2_ degradation in the presence of H_2_O_2_, present in the cells during pancreatic cancer treatment [52]. These nanostructures with activity in SERS and plasmon-enhanced electroreduction were used to detect H_2_O_2_ and uric acid [66].

CuS exhibits a strong LSPR band in the infrared region and thus excellent photothermal, photodynamic, and catalytic properties [97]. Consequently, core–shell PEG-Au@CuS nanostructures were employed in SERS imaging and PTT for colon cancer treatment in mice [67], while Au NRs coated with SiO_2_ and CuS layers showed a significant redshift of the LSPR peak from ∼790 nm (uncoated) to ∼1020 nm. When the core–shell Au@SiO_2_@CuS was functionalized with polycations, the composites were used to deliver antioncogene p53 in a synergistic PTT/gene therapy (GT) breast cancer treatment [67]. In Au@CuS@CuO_2_, Au@CuS determined the good photothermal properties of the nanocomposite, while CuO_2_ generated H_2_O_2_ and Cu^2+^, which reacted with the cellular glutathione (GSH). It was used in the tumor treatment due to the reported sensing and synergetic PTT/CDT capacity [42,43].

### 3.4. Metal-Magnetic Fe_3_O_4_

It has been observed that combining plasmonic metals with Fe_3_O_4_ enhanced the NIR absorption, enabling the resulting composite to be applied in magnetic resonance imaging, and consequently in imaging-guided therapies. For instance, the PTT of Ag@Fe_3_O_4_ nanoparticles was reported to target ovarian cancer cells at 808 nm [68], the core–shell Fe_3_O_4_ @ Au nanostars modified with polyethyleneimine (PEI), PEG, and folic acid (FA) coupled the PTT with magnetic and photoacoustic imaging [69]. Moreover, Zhou et al. designed multifunctional Fe_3_O_4_ @Au hybrids as photothermal agents and photosensitizers for PDT [53]. They correlated the detected singlet oxygen (^1^O_2_) with enhanced plasmonic electron transfer capacity in gold and demonstrated the synergistic PTT/PDT application of Fe_3_O_4_ @Au for the inactivation of cervical cancer cells. Moreover, plasmonic Fe_3_O_4_-based core–shell composites showed good photothermal antibacterial activity under NIR irradiation [98,99]. As reported by Zhao et al., the functionalization of core–shell Fe_3_O_4_ @Au NPs with aptamer enabled the capture, SERS detection, and photothermal inactivation of *S. aureus* [70].

### 3.5. Metal @ Metal

The core–shell architecture is advantageous for bimetallic nanostructures due to its tunable plasmonic properties and improved photothermal stability [100]. Bimetallic core–shell nanomaterials are commonly used as substrates in SERS biosensors to detect biological constituents such as proteins, DNA, and RNA, making them valuable for viral, bacterial, and tumor cells detection [101]. For instance, core–shell Au@Ag nanoparticles coated with a layer of PDA and functionalized with boronic acid were reported to amplify SERS signals by 10^8^-fold and used to detect *S. aureus*, *E. coli*, *S. dysenteriae*, *P. aeruginosa*, and *K. pneumonia* [71]. Also, plasmonic Au@Ag core–shell nanoisland films were used as SERS substrates and for the photothermal inactivation of *E. coli* and *S. aureus* [46]. Au nanorods coated with a Pd shell were functionalized with chitosan and FA and applied for photothermal ablation of tumor cells. Moreover, the bimetallic composite simultaneously generated ˙OH for catalytic therapy and O_2_ for PDT [72]. Core–shell Ag@Au nanoparticles were reported as a catalyst for H_2_O_2_-induced selective oxidative etching of core silver that can be used for the sensing of H_2_O_2_ in vitro [73].

### 3.6. Metal@Organic Macromolecules

Polymers and biomolecules can be used to coat plasmonic nanoparticles, enabling adaptive response to physical and chemical triggers, improved stability, and tunable functionality [102]. For instance, Au@polyaniline (PANI) deposited on graphene oxide was used in a chemo-photothermal cancer therapy coupled with real-time SERS monitoring of DOX release from the core–shell composite [74]. PANI demonstrated good photothermal properties, reduced the toxicity of the plasmonic metal, and produced an intense SERS signal. The synergy between PTT and chemotherapy was based on the hyperthermia determined by the NIR absorption of graphene oxide-Au@PANI and the pH/NIR-sensitive DOX release. Au nanobipyramids@PDA were combined with Au nanoclusters to perform fluorescence-guided PTT [75]. The synergistic performance of Au and PDA in converting NIR light to heat resulted in excellent photothermal properties of Au@PDA. Moreover, PDA showed good photothermal stability for the core–shell nanostructure.

**Figure 6:**
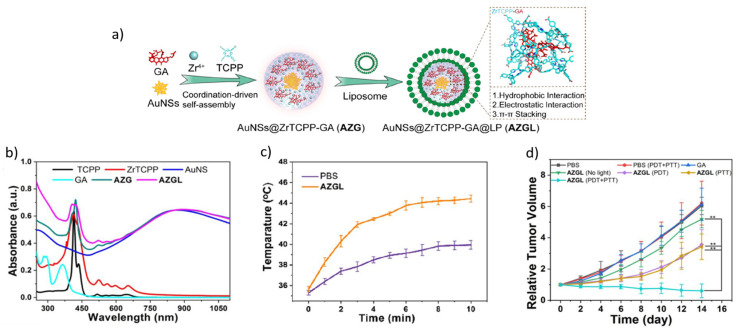
(**a**) Graphic representation of the synthetic route for AZGL nanocomposite formation, (**b**) UV–Vis spectrum of TCPP, ZrTCPP, GA, AuNS, AZG, and AZGL, (**c**) the tumor temperature variation after injection of PBS and AZGL upon 980 nm laser irradiation, and (**d**) tumor growth curves for the investigated cases (*** *p* < 0.001, ** *p* < 0.01, or * *p* < 0.05 were calculated by a Student’s *t*-test). Adapted from [77].

Recently, metal–organic frameworks (MOFs) have attracted significant attention for their high specific surface area, particularly in biomedical applications such as drug delivery systems. For example, Au@Cu_3_(BTC)_2_ NPs were used for Raman imaging and synergistic chemotherapy–PTT (e.g., DOX is loaded on Cu_3_(BTC)_2_). The composite exhibited a higher photothermal efficiency than bare Au, which was attributed to the strong NIR absorption of Cu_3_(BTC)_2_ and its ability to capture the light scattered from the Au core [76]. Also, the synergistic effect of PTT and DOX-based chemotherapy from the core–shell Au nanorods@ZIF-8 nanostructures was an efficient cancer treatment, driven by NIR irradiation-accelerated photothermal decomposition of the ZIF-8 shell and DOX release [24]. Li et al. [77] developed a synergistic therapy for breast cancer, combining PTT–PDT–chemotherapy when using an Au core and a functionalized Zirconium Tetrakis(4-carboxyphenyl)porphyrin (ZrTCPP) shell. As illustrated in Figure 6, Au nanostars @ZrTCPP encapsulated with gambogic acid (GA) and coated with PEGylated liposome (AZGL) degraded in the slightly acidic tumor environment, releasing their components. The synergistic treatment utilized Au-based PTT and PDT, with TCPP acting as a photosensitizer.

### 3.7. Plasmonic Core–Shell Architecture Comparison

The diversity of plasmonic core–shell architectures and their wide-ranging biomedical applications make direct comparisons difficult. Each design offers specific advantages and limitations that depend on the requirements of the intended application, including reaction conditions, irradiation duration, absorption range, biocompatibility, and cost constraints.

Nevertheless, several directions can be identified:

- Silica is preferred as a core component along with Au as a shell. This configuration can be tuned to absorb in the NIR domain when working with spherical particles (more stable and simpler to synthesize).
- CuS is one of the most frequently used semiconductors in plasmonic core–shell structures due to its ability to perform multiple functions in a multifunctional platform.
- Metal@metal composites show strong potential, but their absorption falls mostly in the visible range, making them more suitable for surface applications. To use this architecture in cancer therapies that require NIR absorption, stabilized anisotropic shapes such as nanorods are required.
- MOFs are an attractive component in core–shell nanostructures because they can act as a drug and photosensitizer carrier, with activation triggered by light or by chemical and physical stimuli.
- Polymers have become essential in multifunctional core–shell composites, serving either as a core or as stabilizing shells that reduce toxicity and improve photothermal performance.
- Monitoring the treatment response by SERS, photoacoustic, and magnetic imaging is increasingly important. In this context, Fe_2_O_3_ has been frequently reported as a core material in magnetic imaging-guided therapies.

## 4. Biomedical Applications

### 4.1. Antimicrobial Therapy

Plasmonic nanostructures are a versatile platform for light-activated antimicrobial therapies, offering an alternative to conventional antibiotics for multidrug-resistant pathogens.

#### 4.1.1. Mechanisms of Microbial Inactivation

Plasmonic nanomaterials and nanocomposites convert light into localized heat and generate ROS, enabling synergistic photothermal and photochemical antimicrobial activity through irreversible microbial damage including membrane disruption, protein denaturation, and nucleic acid fragmentation. Therefore, the mechanisms of action (MOA) involve synergistic physical, chemical, and biological vectors to enhance antimicrobial efficacy.

*Photothermal Therapy (PTT).* As mentioned, the primary mechanism is the nonradiative conversion of light into heat via LSPR. Excited “hot electrons” scattered via electron-phonon interactions transfer energy to the metal lattice and subsequently to the environment, thus creating hyperthermia at the NP–microbe interface [103]. Recent transient absorption studies demonstrated that multi-layered core–shell–shell (Au-Ag-Au) architectures exhibited significantly shorter phonon–phonon scattering lifetimes than solid Au nanospheres, leading to superior photothermal conversion efficiency [104]. Additionally, dielectric shells such as SiO_2_ can act as thermal barriers, confining heat within the core and raising the local temperatures by up to 3 times compared to bare particles [105]. The resulting thermal stress destabilizes the bacterial cell membrane, induces protein unfolding, and compromises enzymatic function, leading to cell death.

Importantly, PTT can modulate genetic and biological processes by reversing antibiotic resistance at the genetic level. Heating has been shown to downregulate the fabF gene in *Enterococcus faecalis*, reducing membrane fluidity and re-sensitizing bacteria to Ag^+^ [106]. Furthermore, Ag@ZnO nanocomposites can stimulate the host immune system, inducing keratinocytes to secrete antimicrobial peptides (hBD2, RNase7) and enhancing the lysosomal degradation of intracellular bacteria [107].

*ROS-Mediated Oxidative Stress*: Plasmonic core–shell nanocomposites catalyze the formation of ROS such as hydroxyl radicals, singlet oxygen, and superoxide anions and generate oxidative stress [108]. The reactive species oxidize lipids, proteins, and nucleic acids within microbial cells, thereby amplifying the bactericidal effects and enhancing biofilm penetration. For instance, bimetallic systems such as Au@Pd nanorods exhibite intrinsic peroxidase-like activity, catalyzing endogenous H_2_O_2_ into highly toxic hydroxyl radicals, while simultaneously possessing catalase-like activity to generate oxygen and relieve hypoxia [109]. Also, Reactive Chlorine Species (RCS) represent a novel mechanism using Ag@AgCl nanocubes to generate chlorine free radicals under visible light, which are even more toxic to bacteria than ROS or Ag^+^ alone [110].

*Physical disruption*. Sharp nanostructures, such as AgPd nanodarts, act as “nano-knives,” physically piercing bacterial membranes to facilitate the entry of ions and heat [107,111].

Importantly, beyond single-mechanism structures, the next generation of plasmonic antimicrobials leverages hybrid designs to maximize therapeutic efficiency. By coupling inorganic cores with organic or 2D materials, researchers can engineer “physical coupling” effects that transcend simple addition. For instance, hybridizing Au nanostars with organic dyes such as Indocyanine Green promotes non-radiative decay, boosting the photothermal conversion efficiency to 47.7%—significantly higher than that of bare nanoparticles—allowing for effective bacterial ablation at lower laser powers [111]. Similarly, 2D/0D heterostructures such as MXene-Au@Ag combine the sharp physical edges of 2D sheets with the chemical toxicity of silver ions. In this configuration, the MXene acts not only as a conductive scaffold but also as a “nano-knife” that physically compromises the bacterial envelope, sensitizing the pathogen to the ionic payload [112]. Table 3 presents a synthesis of the synergistic antimicrobial mechanisms of nanocomposites.

#### 4.1.2. Representative Core–Shell and Hybrid Systems

A variety of core–shell and hybrid plasmonic architectures have been explored for antimicrobial therapy. Key examples are summarized in Table 3.

#### 4.1.3. Biofilm Disruption and Therapeutic Advantages

Microbial biofilms represent an important clinical challenge, due to their dense extracellular polymeric substances (EPS) matrix, which limits antibiotic diffusion and makes them inherently resistant. Plasmonic nanostructures offer a unique solution through localized hyperthermia and ROS production, simultaneously disrupting the extracellular matrix and killing embedded cells [113]. Ding et al. [113] created an Au@Ag core–shell system that combines intrinsic chemical toxicity with photo-activated physical disruption. Under NIR femtosecond laser irradiation (820 nm), bacterial-bound aggregates utilize two-photon excitation to generate intense heat and ROS, significantly amplifying the bactericidal effect of released Ag^+^. This multimodal attack is highly effective against resistant phenotypes, achieving a concentration of 7.5 pM against *Staphylococcus aureus* while maintaining high biocompatibility (>88% cell viability). Most notably, this synergy reduced biofilm biomass by 85% within just 4 min of irradiation, a rate unattainable with conventional antibiotics. An example is the treatment of *Enterococcus faecalis* biofilms grown on dentin slices, for which the photothermally activated Au@Ag nanoparticles demonstrated significantly higher bacterial killing rates than 2% Chlorhexidine (CHX), the standard chemical irritant [114]. The mechanism relies on the particles’ physiological behavior: they tend to aggregate in phosphate-buffered saline (PBS) environments, thereby enhancing their NIR absorption cross-section. This aggregation enables intense, localized heating that physically disrupts the biofilm architecture and compromises bacterial cell membranes more effectively than chemical lavage alone [114].

In addition to intensity, incorporating nanostructures into coatings enables scalable surface sterilization. Merkl et al. [123] demonstrated that silver NP aggregates embedded in PDMS could eradicate *S. aureus* and *E. coli* biofilms via NIR-induced localized heating and Ag^+^ release. By using a silica dielectric spacer to tune interparticle distance, the system achieved strong plasmonic coupling in the NIR. Upon 808 nm laser irradiation, these “on-demand” coatings completely eradicated *E. coli* biofilms within 300 s and *S. aureus* within 600 s, providing a potent method for sterilizing medical devices such as catheters.

Consequently, the findings highlight that plasmonic nanocomposites provide a multifaceted approach to biofilm control, targeting both the matrix and the embedded bacteria, and are promising candidates for applications in chronic wound management, implant-associated infections, and surface sterilization. However, the nanocomposite’s structural fine-tuning is one key element of a perfect formulation.

#### 4.1.4. Clinical Relevance

The translation of the research in plasmonic nanocomposites from the laboratory to clinical application is driven by specific advantages over traditional antimicrobials: superior biocompatibility, multimodal deep-tissue mechanisms, and active promotion of wound healing. However, limitations also need to be overcome.

*Biocompatibility and “Green” Synthesis.* A primary barrier to clinical use is the toxicity of surfactants, such as cetyltrimethylammonium bromide (CTAB), commonly used in the synthesis of nanocomposites [108]. To circumvent this, laser-synthesized Si@Au core–satellite systems were fabricated via physical ablation in pure water, eliminating impurities. The silicon core degraded into harmless orthosilicic acid. At the same time, the ultra-small gold satellites (<8 nm) were renally cleared, ensuring the NPs used for the treatment did not persist in the body [110]. Parallel efforts using “green” synthesis with plant extracts have produced stable Au/Ag bimetallics that effectively target resistant strains like *MRSA* and *P. aeruginosa* without hazardous residues [115,116]. Furthermore, geometric optimization allows for precise control over toxicity; tuning the shell thickness of Au@Ag nanoparticles to 5 nm achieved the lowest minimum inhibitory concentration against *S. aureus* while maintaining 99% viability in human cells [117].

*Deep-Tissue Penetration and Hypoxia Relief.* Treating deep-seated infections is complicated by limited light penetration and hypoxic abscesses. Platforms engineered for the NIR-II window (1000–1700 nm), such as AgPd nanodarts, addressed this by absorbing light where tissues are most transparent, achieving record photothermal efficiencies (86.7%) [107]. Simultaneously, hybrid systems such as Au@Pd nanorods relieved hypoxia by acting as artificial enzymes: the Pd shell exhibited catalase-like activity to generate oxygen from endogenous peroxide, thereby refueling the production of toxic radicals even in oxygen-deprived biofilms [72]. The future belongs to innovative thermosensitive platforms, such as the Cu2-xS@MSS-based platform, which exploits the heat generated to trigger the release of loaded antibiotics, delivering a high local dose while minimizing systemic exposure [106].

### 4.2. Cancer Therapy

Cancer remains a leading cause of death worldwide and is challenging to treat due to tumor heterogeneity and the developed resistance mechanisms. The core–shell nanocomposite-based method is a promising approach due to the combined PTT and apoptosis induced by ROS. PTT induces tumor cell death through protein denaturation and mitochondrial dysfunction. According to Melancon et al. [124] and O’Neal et al. [125], PTT can induce either apoptosis for temperatures as high as 41–47 °C or necrosis at higher temperatures of above 50 °C [126,127]. Using NIR-II irradiation allowed for better tissue penetration and efficiency in deep-tissue ablation [124]. This section will focus on ROS therapy mechanisms, tumor-targeted delivery systems using ligands like folic acid and antibodies, and recent advancements in multifunctional nanoplatforms.

#### 4.2.1. Photothermal Ablation Combined with ROS-Induced Apoptosis

To enhance its therapeutic effects, PTT has been combined with ROS-based therapies. This results in a dual cytotoxic mechanism. Hyperthermia increases ROS production by enhancing oxidative stress, leading to mitochondrial apoptosis via cytochrome C release and caspase activation. A moderate heating approach of 42–46 °C for 10 min further enhances ROS diffusion and membrane permeability. In this regard, nanoplatforms of core–shell structure that combine PTT and ROS mechanisms, such as those described in [15,128], have demonstrated better tumor eradication. These platforms support simultaneous drug delivery, real-time imaging, and controlled release, demonstrating significant progress in cancer theranostics [15,129,130,131].

#### 4.2.2. ROS-Based Therapy

ROS-based therapy uses unstable oxygen-derived molecules, such as hydrogen peroxide, superoxide, singlet oxygen, and hydroxyl radicals, to act cytotoxically [132]. While physiological levels of ROS regulate cellular functions, excess ROS causes DNA damage and mitochondrial dysfunction, contributing to apoptosis. Malignant cells intrinsically maintain higher ROS levels, rendering them more vulnerable to oxidative stress-based treatments [133,134]. Various nanomaterials have been designed to take advantage of this vulnerability and cause perturbations in redox homeostasis. ROS can be generated via photochemical, sonochemical, or chemodynamic mechanisms [135]. PDT, using agents such as chlorin e6, generates ROS via light activation, while sonodynamic therapy achieves more effective tissue penetration via ultrasound-activated ROS. Combination approaches, including Ce6-3BP supramolecular systems, have demonstrated improved autophagy and apoptosis [136]. Other platforms, like FHMP NPs, are likewise proposed for image-guided tumor ablation [135].

#### 4.2.3. Targeted Delivery via Tumor-Specific Ligands

Nanocarrier systems have evolved to enable tumor-specific delivery via differential receptor expression. Various ligands, including FA, peptides, aptamers, and monoclonal antibodies (mAbs), improved drug targeting specificity and limited off-target toxicity [137]. FA is one of the widely used targeting moieties because of its high affinity for folate receptors overexpressed in several cancers [138]. The FA-functionalized nanoparticles presented enhanced tumor accumulation and therapeutic indices. Similarly, peptides offer high specificity and penetration, while mAbs enable precise antigen targeting [139]. The functionalized S6 aptamers conjugated to Fe_3_O_4_@Au exemplify targeted PTT, with selective ablation of HER2-positive cells [130]. These nanocarriers are multifunctional, offering ligand-mediated targeting, imaging, and triggered therapeutic release.

Table 4 summarizes applications of core–shell nanocomposites in cancer, while Table 5 presents some cross-studies insights.

### 4.3. Wound Healing and Tissue Regeneration

Wound healing progresses through inflammation, proliferation, angiogenesis, and remodeling, while local infections hinder the process. Conventional antibiotics administered to treat infected wounds are sometimes ineffective due to antibiotic resistance acquired through the misuse in domestic and clinical settings [140]. Therefore, alternatives are sought to reduce the burden on public health [141]. Core–shell nanocomposite systems can make the transition from “killers” (antimicrobials) to “healers” (wound-healing). Core–shell plasmonic nanocomposites accelerate healing by combining antibacterial PTT with photochemical or catalytic ROS generation, oxygen release, and biochemical signaling. For example, Ag@ZnO core–shell particles actively stimulate keratinocyte proliferation, accelerating the re-epithelialization of non-healing ulcers [116]. These systems improve infection control, stimulate fibroblast and endothelial activation, and enhance re-epithelialization and collagen remodeling [142].

Unlike conventional antibiotics, NP-based therapeutic agents interact directly with the bacterial cell wall, do not need to enter the cell, and are less likely to promote bacterial resistance. The intense related research suggested that “core–shell plasmonic nanocomposite for wound healing” is an emerging domain that employes the mechanistic basis of the following synergies:

- Plasmonic cores (Au, Cu_2−x_S) generate heat under NIR irradiation to eliminate bacteria and biofilms,
- Semiconductor or catalytic shells (MnO_2_, CaO_2_, hydroxyapatite) regulate ROS and oxygen levels, promoting angiogenesis,
- Cu^2+^, Ca^2+^, and NO release stimulates fibroblast proliferation and collagen deposition,
- Hydrogel carrier matrices provide moisture retention, adhesion, and programmable therapeutic release [143].

There is growing research supporting the use of core–shell nanostructures combined with hydrogels that merge the PTT and photochemical/photodynamic effects (PDT/ROS generation/chemical kinetics) and demonstrate the ability to treat infected wounds, eradicate bacteria/biofilms, modulate inflammation, and accelerate tissue repair. To facilitate this, not only were the hydrogels loaded with AuAg nanoshells to create photothermal dressings that accelerate the closure of chronic *MRSA* wounds [109], but hybrid photosensitizers such as ZnO@PDA/Ag were embedded in a polyurethane film under NIR irradiation to show strong light-to-heat conversion (photothermal) and generation of singlet oxygen (photodynamic/photochemical). The latter also demonstrated accelerated healing, mature epithelial layer formation, and minimal inflammatory response in a rat wound model [144]. Reviews of the broader field showed increasing interest in photothermal nanosystem scaffolds for tissue regeneration, including composite scaffolds of carbon-, metal-, or polymer-based PTAs (photothermal agents), which—under appropriate conditions—can support tissue repair after infection or injury [145]. Theoretical and experimental studies of core–shell plasmonic nanoparticles have demonstrated that the core–shell geometry often enhances photothermal efficiency relative to bare metal NPs by enabling more efficient heating and tunable plasmon resonances [146]. An enhanced photothermal effect and uniform temperature rise are essential when antimicrobial and tissue-protective actions are required for wound healing. The concept of combining PTT + ROS-based mechanisms (PDT or chemical kinetics)—rather than relying on just heat or just ROS—is increasingly recognized as advantageous, because PTT’s heat can sensitize bacteria or diseased tissue to ROS, and ROS generation can reduce the temperature or dosage needed, reducing collateral damage to healthy tissue [147]. Given that plasmonic core–shell particles have already been developed for PTT, it is scientifically reasonable to consider them for wound healing and tissue regeneration, especially when combined with a scaffold (hydrogel, film) that supports healing, tissue ingrowth, and controlled release. While not strictly core–shell plasmonic NPs, a hydrogel-based system with nanozyme (Ag NPs + polydopamine) that combines photothermal biofilm disruption, responsive ROS-modulating release of antibacterial agents, and ROS-scavenging could be used to shift wound healing away from scarring and toward organized regeneration [148]. However, PDA@Au-HAp NPs, which combined catalysis with NIR photothermal therapy, proved fast, harmless, and effective antibacterial compounds compared to hydroxyl radicals or PTT alone.

Such an assisted nanocatalytic antibacterial system renders bacteria more vulnerable to temperature changes. It demonstrated efficacy against *Escherichia coli* (96.8%) and *Staphylococcus aureus* (95.2%) at a low photo-induced temperature of 45 °C, without causing damage to normal tissues after irradiation with an 808 nm laser. Moreover, the described system enables tissue granulation and collagen production through enhanced tissue-repair-specific gene expression, thereby speeding up the healing process [149]. One resulting composite hydrogel, LPC-Au@HNTs, in a mouse full-thickness skin defect wound model, evidenced excellent photothermal antibacterial activity (*E. coli*—99.00%, *S. aureus*—98.88%), in which Au NPs were sealed into the lumen of Halloysite nanotubes at a surface temperature of 57.59 °C after 5 min NIR irradiation (808 nm) at 1.0 W/cm^2^ [150].

A multifunctional hydrogel comprised of one contractable lower thermoresponsive poly(N-isopropylacrylamide)/gelatin methacrylate layer and an upper highly stretchable alginate/polyacrylamide layer embedding different peptide-functionalized gold nanorods (AuNRs) in each layer promoted a two-stage sequential release of embedded cargos during the early stages of wound healing. The synergism upon NIR stimulation demonstrated antibacterial and pro-angiogenic efficacy [151].

Also, a composite hydrogel system of polydopamine–hyaluronic acid (PDA-HA) hydrogel-loaded calcium peroxide–indocyanine green combined with lauric acid and manganese dioxide (CaO_2_-ICG@LA@MnO_2_) nanoparticles showed the synergism between excellent photothermal performance under NIR irradiation and controllable and sustainable release of oxygen and ROS for controlled inflammation, tissue regeneration, and repair in chronic wounds [152].

CuS nanodots were synthesized directly inside a gelatin host matrix (Gel-CuS), with excellent dispersibility and stability against oxidation, then crosslinked with oxidized dextran (ODex) to form a Gel-CuS-8/ODex hydrogel (8 stands for the concentration of CuS, in mM) with improved mechanical properties, excellent adhesion and self-healing ability, suitable swelling and degradation behavior, and good biocompatibility. The hydrogel acted as an effective antibacterial mediator due to its photothermal and photodynamic properties under a 1064 nm laser exposure and, in animal experiments, as wound dressing, significantly promoting full-thickness cutaneous wound healing after NIR therapy [153].

Compared to hydrogel dressings with individual active agents (CuS Gel or NO Gel), CuS/NO Gel demonstrated better antibacterial and repair-promoting activity in vitro and in vivo, along with good mechanical stability, high photothermal activity, and excellent biocompatibility. The effects were due to synergism: photothermal and NO release in the infection phase, and enhanced vascularization and collagen deposition due to the increased contributions of Cu ion nutrients and low-concentration NO signaling molecules [154].

The related research so far evidenced specific advantages of core–shell structures over bare NPs and supports the future development and applications because of:

- more efficient and controllable heating under light irradiation [146].
- the ability to combine multiple functionalities—e.g., a metal core for heat + a shell that carries or facilitates ROS generation, drug release, or catalytic activity [155].
- the potential for improved stability, biocompatibility, and controlled interactions with the biological environment—all of which are critical for wound healing applications [145].

While core–shell plasmonic nanocomposites offer multifunctional advantages for wound healing (antimicrobial action, photothermal therapy, controlled delivery), their clinical success depends on overcoming challenges related to toxicity, thermal control, stability, immune response, scalability, and regulation.

**Figure 7:**
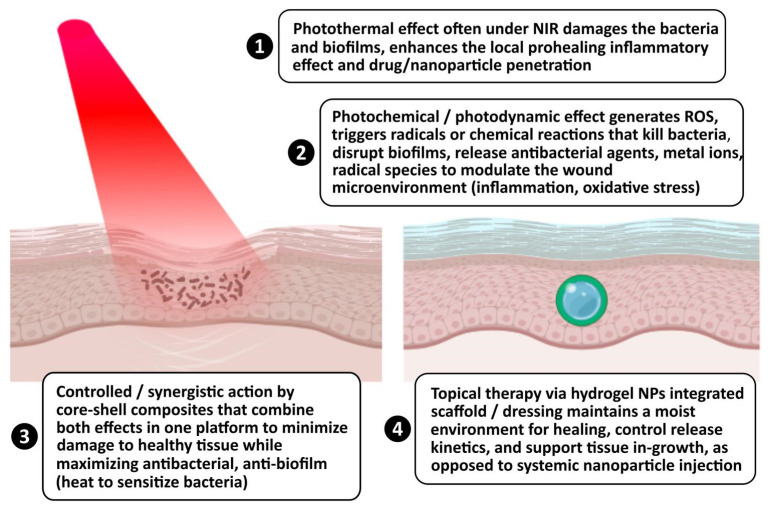
The synergy between photothermal + photochemical core–shell nanocomposite for wound healing.

In conclusion, the published studies showed that plasmonic/photothermal + photodynamic/photochemical nanocomposites can enhance wound healing and tissue regeneration after biofilm-associated infection. Core–shell NP architectures are ideal for such applications due to their tunable plasmonic properties, enhanced photothermal conversion, and support for chemical/photochemical functionalities. Figure 7 summarizes why “synergistic photothermal + photochemical core–shell nanocomposite for wound healing and tissue regeneration” presents real clinical potential.

### 4.4. Biosensing and Bioimaging

Core–shell plasmonic nanocomposites have emerged as powerful tools in biosensing and bioimaging due to their unique optical properties, particularly their ability to enhance light–matter interactions through LSPR [156]. These properties enable advanced techniques such as SERS, fluorescence, and photoacoustic imaging, which offer high sensitivity, specificity, and spatial resolution for biomedical diagnostics. Additionally, ROS or thermal feedback mechanisms can be integrated into detection systems, further enhancing their functionality. A comprehensive summary is provided in Table 6.

#### 4.4.1. Surface-Enhanced Raman Scattering (SERS)

SERS leverages the strong electromagnetic field enhancement near plasmonic nanoparticle surfaces (e.g., Au, Ag cores) to amplify Raman scattering signals from biomolecules [157]. Core–shell architectures, such as Au@SiO_2_ or Ag@TiO_2_, improve SERS performance by, for example, enhancing the signal intensity. In this way, the plasmonic core amplifies the local electromagnetic field, increasing the sensitivity for detecting low-concentration analytes like proteins, DNA, or small metabolites. Improving the stability can also lead to better performance. The shell (e.g., SiO_2_, polymers) protects the core from aggregation and environmental degradation, ensuring reproducible signals. Also, functionalization can be an option for improvement in performance. Shell surfaces can be modified with biorecognition elements (e.g., aptamers, antibodies) for selective analyte binding, enabling applications like cancer biomarker detection or pathogen identification. For example, Au@SiO_2_ nanoparticles functionalized with Raman reporter molecules have been used for ultrasensitive detection of circulating tumor cells, achieving detection limits down to single-cell levels [158].

#### 4.4.2. Fluorescence-Based Imaging

Core–shell plasmonic nanocomposites enhance fluorescence imaging by exploiting plasmon-enhanced fluorescence (PEF). The LSPR of the metal core (e.g., Au, Ag) can increase the excitation efficiency of nearby fluorophores or quench fluorescence depending on the core–shell distance. There are a few advantages to consider: firstly, the ability to tune the emissions by adjusting shell thickness (e.g., in Au@polymer or Au@SiO_2_ systems) or the distance between the core and fluorophore, which can be optimized to maximize emission intensity. It is also possible to enhance fluorescence signals to improve imaging contrast, enabling visualization of cellular structures or tissue-level processes, while different core–shell compositions can support multiple fluorophores, facilitating simultaneous imaging of multiple biomarkers. For instance, Au/ZnO nanocomposites have been used to enhance fluorescence imaging of tumor tissues, combining high brightness with photostability for real-time monitoring [159].

#### 4.4.3. Photoacoustic Imaging

Photoacoustic imaging relies on the conversion of absorbed light into acoustic waves, offering deep tissue penetration and high spatial resolution. Core–shell plasmonic nanocomposites, particularly those with strong NIR absorption (e.g., Au@carbon or Au@TiO_2_), are ideal for this modality. This is due to their high photothermal conversion efficiency, as the plasmonic core absorbs light and generates heat, producing strong acoustic signals. It is also due to their tunable absorption capacity, given that the LSPR tuning into the NIR window (700–1100 nm) enables deeper tissue penetration, critical for in vivo imaging. Based on these characteristics, photoacoustic imaging has theranostic potential, as it can be combined with PTT (NPs can generate diagnostic signals and heat). An example is Au@SiO_2_ nanoparticles used for photoacoustic imaging of tumors, where the silica shell enhances biocompatibility and allows functionalization with targeting ligands [160].

#### 4.4.4. ROS and Thermal Feedback-Based Detection Systems

Core–shell nanocomposites can integrate ROS or thermal feedback for dynamic biosensing. Photocatalytic shells (e.g., TiO_2_, ZnO) generate ROS under light irradiation, which can be used to detect oxidative stress or monitor biomolecular interactions. For example, ROS generation in Au@TiO_2_ nanosystems has been exploited for glucose sensing, where ROS reacts with glucose to produce detectable byproducts [161]. Photothermal effects from the plasmonic core can modulate sensor responses, such as in temperature-sensitive fluorescence or SERS probes. This is particularly useful for real-time monitoring of cellular microenvironments or drug release [162,163].

**Table 6:** Examples of core–shell systems used in biosensing and bioimaging.

Material	Applications	Wavelength	Observations	Ref.
SPION-PEI@AuNPs	SERS for ultrasensitive detection of circulating tumor cells, cancer biomarkers	LSPR-tuned (visible–NIR)	Enhances electromagnetic field; shell provides stability and functionalization with aptamers/antibodies (e.g., FA-conjugated rBSA); detection limits to 1 cell/mL	[154,158]
Ag@TiO_2_	SERS for detecting proteins, DNA, small metabolites, pathogens	LSPR-tuned (visible–NIR)	Strong field enhancement; improves signal intensity and reproducibility; protects core from aggregation (supported by multiple studies on Ag/TiO_2_ hybrids for biomolecule SERS)	[164]
Au@Ag@SiO_2_	Fluorescence-based imaging of cellular structures, tumor tissues	Tunable via shell thickness (visible–NIR)	PEF; optimizes emission intensity and contrast; supports multiple fluorophores for multi-biomarker imaging	[165]
Au/ZnO	Fluorescence imaging with radiosensitization for triple-negative breast cancer	Tunable (visible–NIR)	High brightness and photostability; modulates tumor hypoxia and ROS generation	[159]
Au@carbon/Au@TiO_2_	Photoacoustic imaging of tumors	NIR (700–1100 nm)	High photothermal conversion efficiency; deep tissue penetration; theranostic potential combining diagnostics and therapy (supported by carbon-plasmonic photoacoustic agents; for similar Au@SiO_2_)	[166]
Au@SiO_2_ (functionalized)	Fluorescence imaging with targeting	NIR (700–1100 nm)	Silica shell enhances biocompatibility; allows ligand attachment for specificity	[160]
Au@TiO_2_	ROS-based glucose sensing, oxidative stress detection	Light irradiation (UV–visible)	Photocatalytic shell generates ROS; modulates sensor responses via photothermal effects	[161]
SiO_2_/ZnO shells	Thermal feedback in dynamic biosensing, drug release monitoring	Light irradiation (UV–visible–NIR)	Integrates ROS/heat for real-time cellular microenvironment monitoring	[162,163]
Magnetic–plasmonic hybrids	MRI-guided cancer detection/treatment, magnetic hyperthermia	NIR-II (1000–1700 nm)	Enables deeper penetration, reduced photodamage; synergy with targeting and therapy	[167]
All-organic/biodegradable core–shell	Stimuli-responsive theranostics (pH/enzyme/light-triggered ROS/heat release)	NIR-II (1000–1700 nm)	Addresses toxicity concerns; integrates with immunotherapy/gene editing for precision medicine	[168]

## 5. Design Considerations and Performance Optimization

**Figure 8:**
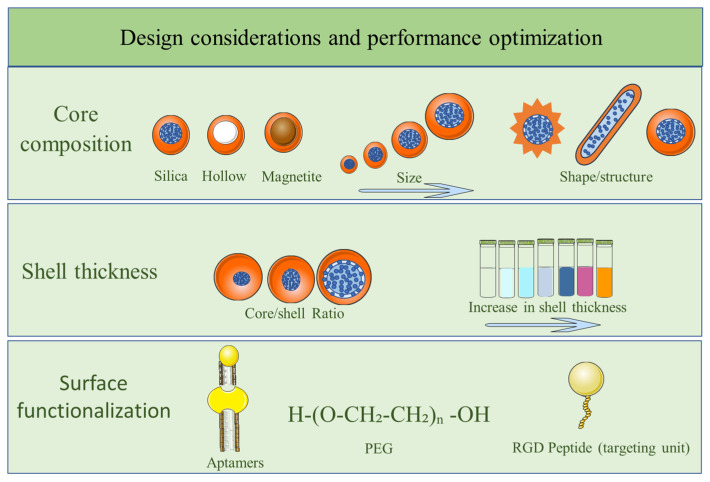
The design of nanostructures with different structures and shapes to tailor the LSPR efficacy. The arrows indicate the increase in the size of the core-shell NP and increase in the shell thickness of nanoshells.

Given the necessity to meet the demands of large absorption cross sections, outstanding stability, and less cytotoxicity, core–shell nanomaterials with plasmonic features offer a versatile platform to mitigate the challenges associated with single-component nanostructures and bulk materials. This fact, in addition to ensuring easy post-modifications while still promising reproducibility, thus achieves optimal photothermal and photochemical performance, especially for biomedical applications. Therefore, attaining this requires intricate design and fabrication of core–shell structures with novel LSPR-enhanced optical and catalytic functionalities, primarily through meticulous optimization across material, structural engineering, surface functionalization, and biomedical compatibility (as summarized in Figure 8).

### 5.1. Core Material Selection Based on LSPR Range and Biocompatibility

The design and fabrication of plasmonic core–shell nanostructures require the selection of a core material that possesses strong optical response, low cytotoxicity, and the ability to cause photothermal light-to-heat conversion. NPs made of Au are the most widely used core for in vivo applications due to their remarkable stability and intense, tunable LSPR across the UV to the NIR range, in addition to their ease of functionalization, chemical inertness, and low cytotoxicity [169,170]. Of particular interest are the two biological windows of NIR-1 (650–900 nm) and NIR-II (1000–1400 nm) due to their generation of elevated heat, which reduces absorption and scattering of photons by nearby tissues and significantly accelerates chemical reactions, facilitating deep tissue penetration and treatment. Considerable effort has been devoted to exploring advanced methods to amplify the performance of Au-based nanosystems by structuring them at the nanoscale. For instance, when Au metal was combined with a Cu_2−x_Se semiconductor into a core–shell hybrid, the absorbance of Au@Cu_2−x_Se at 808 nm laser at 1.0 W cm^−2^ was much superior to that of Au NPs, Cu_2−x_Se NPs, and the physical mixture of Au NPs and Cu_2−x_Se NPs. The enhanced LSPR intensity of Au@Cu_2−x_Se facilitated heat generation for the destruction of tumor growth [171]. Concomitantly, Cai et al. [42] designed a core–shell nanostructure by growing CuS on gold NPs (AuNPs) and subsequently modified the surface of Au@CuS (AC) with CuO_2_ to produce ACC NPs. Compared with using a single Au NPs, the ACC nanoplatform achieved a redshift in its light absorption range from visible to near infrared (300 to 1200 nm) and showed higher temperature solution increases under irradiation with an 808 nm laser, which is efficient as breast cancer treatment. Along similar lines, Chang et al. [172] focused on designing a plasmonic yolk–shell nanostructure with enhanced PTT/PDT by activating resonance energy transfer. The metal core@void@semiconductor shell yolk shell nanostructure was designed by employing an Au core with a different LSPR absorption peak and a template method based on Au@Cu_2_O core–shell NPs. The surface of Au@Cu_2_O was transformed into a CuS shell by reacting with sodium sulfide, after which the surface was fabricated with thermo-responsive thiol-terminated polymer (P(NIPAM-*co*-AM) to activate controlled drug release. This method leverages not only the LSPR overlapping of Au and CuS but also the PIRET process to significantly improve the LSPR absorption on Au-CuS YSNPs compared to that of CuS NPs. Under 808 and 908 nm irradiation at a density of 1 W cm^−2^, the synthesized Au-CuS YSNPs triggered the highest temperature elevation, with the most potent photothermal conversion achieved through tuning the LSPR peak of the Au core. Meanwhile, the activated Resonant Energy Transfer (RET) promoted more hole formation in the CuS shell, yielding more OH radicals during NIR irradiation, suggesting synergy between PTT and PDT. Interestingly, the prepared Au-CuS YSNPs were non-toxic to 4TI cancer cells but lethal upon 808 nm laser irradiation, and they showed promising results in vivo for cancer therapy.

The design of nanostructures with diverse shapes and structures is another way to tailor the LSPR frequencies in the two biological NIR windows, of NIR-1 and NIR-II, thereby facilitating deep tissue penetration and treatment [173]. Typically, non-spherical shapes, such as rod-like forms, exhibited more resonance directions than the spherical analogues. Besides forming additional peaks and redshifts, the former exhibit significant near-field enhancement under similar volume conditions as opposed to the latter, as shape variations enabled the capture of a wide range of NIR desired for biomedical applications [169]. Since the optical behavior can be controlled through changes in the geometry of NPs, different NPs configurations—including Au nanorods (AuNRs), silica-Au nanoshells, and Au nanospheres (AuNSs)—were compared for their shape-dependent effect on their light-absorption capacity. Their results showed that the values of the volumetric absorption and scattering coefficients for AuNRs were superior to those of nanospheres and the nanoshells. This property of AuNRs allows them to exhibit more NIR light absorption and scattering efficiency. For smaller volumes and higher aspect ratios, AuNRs showed higher photoabsorption efficiency, whereas increasing volume increased light-scattering efficiency [174]. Moreover, shape-dependent variables affected the heat generation of plasmonic NPs, as enhanced light-absorption properties fundamentally drove the production of photothermal effects. For example, the heating efficiency of illuminated AuNPs as a function of morphology revealed that AuNRs, when compared with AuNSs of the same size, generated plasmonic resonance, which enhanced the heat conversion efficiency of the nanostructures. The authors attributed the heat generation to the longitudinal plasmon modes on the rods, which redshifted and led to a substantial increase in heating efficiency [175]. The main merit of anisotropic plasmonic nanostructures, as compared to other nanomaterials, is the tunability of the SPR phenomenon in a broad wavelength range. It is well known that gold nanoshells exert a PTT effect upon absorption of NIR light [176]. Vankayala et al. [177] implemented Au nanoshells (including nanocages, nanorods in shell, and nanoparticle-in-shell) for bimodal PDT/PTT cancer treatment. By tuning the gold nanoshell NPs, LSPR red-shifting was induced and reached the NIR region, which is necessary to trigger cell death. Interestingly, benefiting from the plasmonic photothermal effect combined with singlet oxygen generation, the nanostructures effectively destroyed B16F0 tumor cells under low doses of NIR light (980 nm) irradiation, which is not toxic to surrounding normal tissues. In contrast to the NP-in-shell, manipulating the excitation wavelength primarily induced PDT for the nanocages and the nano-rod-in-shell to induce cell death.

As mentioned above, precisely tuning the size and shape of plasmonic core–shell NPs optimized performance by shifting the position and height of the LSPR absorption peaks. As the particle size increased, the resonance wavelength redshifted due to the generation of hot electrons near the Fermi level; smaller particles have higher energy levels, enabling them to overcome the Schottky barrier more effectively and promoting plasmon-induced catalytic reactions [178]. In general, smaller NPs served as photoabsorbing agents for biomedical applications due to their higher surface area-to-volume ratio. In comparison, larger NPs were preferred for biological imaging owing to the dominant role of scattering effects [179]. Yang et al. [180] explored the influence of Au satellite size on plasmonic coupling by growing different Au NPs as satellites with tailored sizes into an Au@Cu_2_O@Au core–shell satellite structure for enhanced photocatalysis. They experimentally and theoretically demonstrated that precise tuning of the Au NPs induced modal splitting in the extinction spectra, suggesting the formation of modal coupling. The first increase in the Au satellite resulted in a gradual broadening of the dipole resonance peak of the Au@Cu_2_O@Au, and a shoulder peak (~600 nm) appeared when further increased, suggesting that dual-extinction bands due to peak splitting induced by modal coupling were formed. As the shoulder peak becomes stronger, a broad extinction band was observed, indicating the modal coupling between the nanocavity mode and the LSPR modes of the Au satellites. Therefore, their work highlighted that tuning the structural features and plasmon coupling of the Au@Cu_2_O@Au NPs by controlling their size is indispensable for optimizing optical properties and enhancing synergistic photochemical reactions via plasmon-induced hot carriers.

Beyond Au, Ag and Cu are other types of promising nanomaterials with LSPR properties that resonate in the visible light range, with Ag displaying a stronger plasmon response than Au. The interest in Ag NPs for surface-based disinfection and biosensing stems from their ability to convert light to heat through photothermal effects, leveraging their optical features, including absorption, scattering, and enhanced extinction [181]. However, since Ag is considered toxic for in vivo applications and is prone to oxidation and ion leaching, addressing this shortcoming requires novel designs to improve its biocompatibility and stability in physiological environments. For the optimal observation of this effect, Lin et al. [182] proposed a nanosystem based on a hollow silver–gold alloy immobilized with chlorin e6 (Ag@Au-Ce6), a photosensitizer for killing bacteria and ameliorating wound healing. By tuning the concentration of Ag@Au-Ce6 NPs, they were able to induce a strong LSPR effect in the near infrared region, resulting in increased temperature for each concentration at 46.5, 51.5, 55.1, and 57.5 °C upon exposure to an 808 nm laser for 5 min at a power of 0.8 W cm^−2^. The designed Ag@Au-Ce6 nanostructure displayed promising results in generating excessive ROS within the bacteria after exposure to laser irradiation, resulting in a decline in the survival ability of the *S. aureus* and *E. coli*, and eventually leading to death. In this way, these authors established that this dual action of PTT/PDT underscores the potential of plasmonic Ag@Au-Ce6 to drive photochemical reactions, enabling favorable performance in the healing of bacteria-infected wounds. On the other hand, low-cost copper and copper sulfide (Cu_2−x_S) plasmonic cores are capable of providing strong absorption in NIR-I and NIR-II, with the added benefit of Cu^+^/Cu^2+^ ions enhancing antimicrobial activity. In addition, the biodegradability of Cu is superior to that of noble metals commonly used in cancer therapy [183,184]. Driven by the electromagnetic field of incident NIR light, these semiconductors also possess metal-like plasmonic features that originated from the resonantly excited free hole oscillations in the valence band. The tunable NIR optical resonance of Cu_2−x_S, in combination with the generation of high levels of ROS, produced material with improved overall performance for broader biomedical applications. For example, Wang et al. elucidated the impact of Cu_2−x_S nanocrystals on plasmon-driven PTT and PDT therapeutic effects in cultured melanoma cells and a murine melanoma model [185]. The Cu_2−x_S therapeutic agent elicited combined action of PDT and PTT by extinguishing NIR laser light and dissipating the absorbed photon energy as heat. By engineering the LSPR energy of copper sulfide NCs via redox reactions, the desired optical properties were achieved through their chemical composition. Interestingly, NIR laser light triggered enhanced ROS generation and elevated temperature due to leaked copper ions from copper sulfide. The effect of the increase in the free charge carrier density was observed by the shift in the LSPR to higher energies due to the additional holes in the upper edge of the valence band introduced by oxidation. Moreover, the prepared plasmonic nanocomposite ablated tumor cells through a substantial dual PTT/PDT effect under NIR with minimal toxicity and deep penetration.

In addition to manipulating the size and structure of nanoplasmons, the dielectric properties of the core play a crucial role in optimizing the nanomaterial’s optical response and the electric field distribution. Based on the differing dielectric properties of different materials associated with the refractive index, various plasmonic materials, in fact, revealed different LSPR wavelengths. Core materials with a real component of the dielectric constant responded with a redshift, whereas the imaginary part reduced the intensity of the resonant wavelength and resulted in a decrease in surface plasmon coupling. Furthermore, achieving a decrease in the cavity-plasmon dampening and an increase in LSPR spectral intensity involved adding a nonabsorbing material with a real refractive index, such as silica [186]. Meanwhile, to further improve the performance of Au nanoshells, Sikdar et al. compared gold nanoshells with different dielectric fillings and found that hollow nanoshells with low absorption efficiency were preferable to the strongly absorbing ones, as they are not detrimental to neighboring healthy tissues [187].

Engineering plasmonic nanomaterials comprising a magnetic core and a functional shell to achieve a strong optical response has attracted attention in the biomedical field. This fact is attributed to the magnetic core’s ability to deliver nanocarriers to the specific target site due to its superparamagnetic properties while contributing to the physicochemical features of core–shell nanocarriers through their tunable properties [18]. Attaining tunable plasmonic effects from a magnetic core with functional integration required intricate tailoring within the tissue-transparent region. Towards achieving synergistic enhancement effects for application in cancer therapy, the concept of absorbing gold nanopopcorns with an iron oxide cluster (IOC) core for magnetically amplified PTT/PDT treatment with 11-mercaptoundecanoic acid (MUA)-PEG coating was explored. Interestingly, the IOC-Au NPCs paired with electromagnetic radiation and displayed strong light-to-heat conversion efficiency, with a broad absorption peak around 800 nm. Compared to the IOCs, the diamagnetic properties of Au contributed to the enhanced superparamagnetic behavior of the IOC-Au NPCs, with a saturation magnetization of around 22 emu/g, which is desirable for deep penetration of the nanocarrier and accelerated drug release. The study showed that even at low doses of the formulated drugs and low laser intensity (0.55W/cm^2^), SK-BR-3 cells were eradicated under combinatorial PTT and PDT in conjunction with magnetic-field-guided drug delivery using the nanocomplex [188].

### 5.2. Shell Thickness Optimization for Efficient Energy Transfer and Permeability

Beyond passive coating, the shell, comprising different materials (metals or dielectrics) in a core–shell structure, served as an effective thermal protective layer for the core while controlling the distance and alignment between plasmonic sites and the active positions. These features, in addition to the regulation of water, O_2_, substrates, and analyte permeability, combined with the control of charge separation and hot-electron extraction, can influence stability and dispersibility of the cores, protect them from oxidation or degradation, and offer a platform for tailored functionalization of the cores to meet specific application requirements [63]. To boost the performance of NPs in biological environments, the LSPR can be shifted to longer wavelengths by manipulating the shell thickness [189]. According to simulation studies, the plasmonic response of silica–gold nanoshells can be tuned by adjusting the core radius ratio to the shell thickness. Significant absorption under NIR excitation becomes markedly stronger when thin shells in the 5–15 nm range are used on relatively large cores and heat transfer to the surrounding medium is accelerated [33]. To elucidate the shell-dependent influences on the extinction spectra, Lui et al. [190] created a core–shell nanoparticle comprising Au and Cu_2_O (Au@Cu_2_O) to enhance the LSPR extinction of the nanoparticles. Their results indicated that by precisely controlling the shell thickness, the nanoparticles displayed a significant red shift of the SPR (dipole band) in the near-infrared region, with enlarged optical cross sections. Interestingly, the theoretical modeling matches the experimental spectra, which showed clearly enhanced intensity and peak position of the LSPR band, resulting in improved light absorption. The ability to tune the LSPR by adjusting the shell thickness in Cu_2_O offers an approach to efficiently harness the full spectral range, demonstrating strong potential for biologically relevant applications.

### 5.3. Stability Under Physiological Conditions

When biological applications are considered, a key practical bottleneck is maintaining the colloidal and chemical stability of core–shell particles in physiological media, including PBS, serum, cell culture, and in vivo. High ionic strength and abundant proteins tend to induce aggregation, opsonization, and changes in surface charge. Given the impact of unstable nanomaterials in SPR absorption loss, PEG coatings remained the most robust technique not only to provide steric stabilization and protection from salt-induced aggregation but also to reduce protein adsorption and immune recognition [191]. Hydrophilic PEG chains with a flexible backbone can covalently form strong bonds when grafted on the surface of NPs through various reactive groups such as amines, thiols, and carboxyl functions [192]. Representatively, Ahmad et al. [193] designed PEG-coated gold-doped titania with a mutual photothermal/photodynamic effect targeting MCF-7 cancerous cells. A wide range of electromagnetic field development was observed by varying the nanostructure morphologies, indicating a better utilization of visible light via LSPR to maximize cancerous cell injury in the MCF-7 cell line. By increasing the concentration of PEG-coated Au-doped titania, cell viability was significantly reduced, demonstrating remarkable drug delivery towards the targeted/cancerous site. Additionally, integrating PEG into the system significantly improved biodistribution and therapeutic efficacy. Moreover, core–shell particles with surface molecularly imprinted polymer (MIP) shells further improved the dispersibility, storage, and colloidal stability in biological fluids, while the plasmonic core provided optical transduction [194].

### 5.4. Surface Functionalization with PEG, Peptides, or Aptamers for Targeting and Stealth

Beyond generic stabilization, strategies such as surface functionalization defined how core–shell nanocomposites interact with cells, tissues, and pathogens. The modified core–shell provides new functions by virtue of its efficiency in integrating materials with complementary functions for biomedical applications. In the exploration of near-field enhancement through the functionalization of plasmonic nanostructures, various compounds, including PEG, peptides, and aptamers, have been applied to provide the desired properties for biomedical applications [195]. The attractive attributes of PEG, including reduced opsonization, extended circulation half-life, and minimized uptake by the reticuloendothelial system, are beneficial for tumor-targeted PTT/PCT and biosensing. For example, there is compelling evidence that PDT/PTT is highly potentiated by the presence of PEG, ultimately enhancing the biocompatibility and tumor accumulation of core–shell nanostructures.Therefore, fluorescein-labeled and thiol-functionalized PEG were used to modify Pd@Pt nanocubes to acquire an LSPR platform with enhanced PTT/PDT therapy of hypoxic tumors. Interestingly, the Pd@Pt-PEG nanocubes achieved excellent biocompatibility and inhibited the tumor cells’ growth. In the meantime, the optical response of the excitation plasmons increased, leading to substantial hyperthermia and further inducing the formation of singlet oxygen due to the deposited Pt shell on the Pd core [196]. Aptamers are short, single-stranded nucleic acids that can also be attached to plasmonic NPs for selective targeting of cancer cells, SERS or LSPR biosensing with molecular recognition, and early detection and treatment of diseased cells. Owing to the strong affinity and high targeting specificity of aptamers, broad reviews have proposed aptamers as targeting moieties for delivering therapeutic agents in cancer gene therapy, thereby achieving optimal treatment specificity and efficacy. Experimental evidence supports the use of aptamers as a switch probe to modify AuNRs for the delivery of chlorin e6-polyvinylpyrrolidone in PTT/PDT cancer treatment. The attachment of the nanosystem to cancer cells induces the release of chlorin e6 from the surface and, thus, the generated ROS upon light irradiation eliminated cancer cells. The gap distance between a quencher and a photosensitizer was varied to control ROS generation via aptamer switches, enabling the quenching and recovery of photosensitizer fluorescence [197]. Meanwhile, peptides are another notable targeting ligand that offer several advantages, such as good stability, and they confer biological functionality for nanoparticle-based therapy [198]. While being synthetically accessible and programmable, they have high interaction affinity and selectivity for specific receptors on cancer cells and can be easily conjugated to nanoparticles. Therefore, peptide-modified core–shell nanoparticles, with the appropriate size and physicochemical features combined with surface chemistry, enabled active targeting at the appropriate time for effective therapy [199]. For example, tumor-homing peptides, such as cyclic arginine–glycine–aspartic acid (cRGD), when conjugated to Au@Pt plasmon NPs, facilitated active targeting and increased selective uptake [200]. To obtain DOX/Au@Pt-cRGD, a PEG linker was coated on the surface of the Au@Pt core–shell to serve as a support for the incorporation of cRGD, which endowed the DOX/Au@Pt-cRGD with colloidal stability and anticancer therapeutic effects. The peak of absorbance of the Au@Pt system in the NIR window offers the potential for in vivo photoacoustic imaging and a high photoconversion efficiency, highlighting the potential of Au@Pt nanoparticles as a suitable carrier for PTT.

## 6. In Vitro and In Vivo Toxicity

### 6.1. General Aspects

Based on cells and organisms affected by the engineered layers (core, shell, ligands), the toxicity of core–shell plasmonic nanocomposites is system-dependent. Because the main plasmonic-type core–shell systems (Au@SiO_2_ (gold core, silica shell), Au@Ag or Ag@Au (gold–silver bimetallic), magnetic–plasmonic (FePt@Fe_2_O_3_@Au or iron oxide@gold)) serve many applications (SERS, imaging, photothermal therapy, drug delivery, sensing), all toxicity data come from biomedical and environmental studies. Structures and functions are distinct key determinants, it is observed [167]. As for gold, a biocompatible metal, when reasonably sized and coated, the core–shell design can lower the toxicity of bare AuNPs [201]. Studies on core–shell architectures demonstrated that a silica coating on Au nanostars or nanorods mitigated direct membrane interaction and often reduced toxicity. However, a CTAB template can remain within the silica, causing significant cytotoxicity unless adequately removed or replaced [202]. In human breast cancer cells, gold–mesoporous silica core–shell particles exhibited lower cytotoxicity than bare Au, and Janus Au@mSiO_2_ was the least toxic among the tested formulations. In addition, amorphous silica (SiO_2_) shells prevented aggregation and contributed the functional groups. Studies have shown that amorphous silica coatings reduce toxicity and increase biocompatibility when compared to bare Ag or bare Au [203]. Although toxicity is strongly size-, porosity-, and surface-chemistry-dependent for silica NPs, a recent safety review observes that thin, homogeneous silica shells remain more biocompatible than bare metallic surfaces, particularly when PEGylated. Recent reviews on magnetic–plasmonic core–shell NPs (Fe_3_O_4_@Au, Au@Fe_3_O_4_, organosilica shells) demonstrated that PEGylated and thiolated organosilica shells exhibited acceptable in vitro and in vivo toxicity at therapeutic doses. In contrast, unmodified or cationic surfaces are less well tolerated [204]. Magnetic–plasmonic core–shells with good coatings (Fe_3_O_4_, FePt cores often PEGylated and/or silica-coated) presented good in vivo toxicity profiles at therapeutic doses, with effects varying according to coating and dose [167].

In contrast, the toxicity of silver (Ag) was associated with the liberation of Ag^+^ ions, leading to oxidative stress, DNA damage, mitochondrial dysfunction, and inflammation [205]. The toxicity of Au@Ag or Ag@Au systems is due to the amount of silver and the rate at which the silver is dissolved. Membrane damage and enzyme blockade were the result of dissolved ions, in particular Ag^+^ [206]. Green-synthesized Ag@Au NPs (plant extract) demonstrated significant anticancer cytotoxicity against HeLa cells, with an IC_50_ in the tens of µg/mL, suggesting high potency/overall toxicity [207]. Bimetallic Ag@Au NPs exhibit composition-dependent cytotoxicity and anti-bacterial activity, which means that a higher concentration of Ag will enhance antimicrobial and anticancer effects at the cost of greater toxicity towards mammalian cells [208]. Au@Ag has greater toxicity against tumor cells than bare Au, and against normal cells if not cautiously adjusted [209]. Alginate-stabilized Au@Ag nanostructures led to reduced sperm parameters, testicular damage, and reduced fertility in an in vivo model associated with oxidative stress and Ag content [210]. The studies found that Ag^+^ release and ROS generation remain serious challenges, despite the Au core [211]. Synthesized Ag/Au core–shell nanoparticles from seed growth and their application in human blood components exhibit hemolytic and other hematological changes, emphasizing that even though Ag–Au is prepared with a noble-metal shell, it can be hemotoxic at relevant doses [212]. Au nanorod core @ Ag shell @ mesoporous silica (AuNR@Ag@mSiO_2_) or similar multilayer plasmonic platforms for SERS and phototherapy exhibit excellent in vitro properties, but the results need careful cytotoxicity and hemocompatibility characterization. The generation of ROS under irradiation, in addition to being a feature (for therapy), is also a safety concern [213].

The most important determinants of the biocompatibility of core–shell plasmonic nanocomposites are size, shape, and surface charge. For example, small NPs possess a larger surface area, can cross biological barriers, and in many cases exhibit enhanced reactivity and toxicity [214]. At high doses and without protective shielding, AuNPs < ~50 nm induce DNA damage and other cytotoxic effects via oxidative stress and nuclear interactions [215]. Moreover, spherical Au NPs are frequently reported to be more toxic than rods, stars, or other shapes at equal mass doses, attributed to differences in cellular uptake and surface area [216]. Very small (<10 nm) or high aspect ratio/sharp-tip particles (nanorods, nanostars) are more likely to penetrate membranes and organelles [217].

At the same time, positively charged surfaces (e.g., amine, PEI) generally cause greater membrane damage and higher cytotoxicity than neutral/PEGylated or negatively charged surfaces. PEGylation and biocompatible polymers generally reduce protein adsorption, aggregation, and immune activation, thereby lowering toxicity [214]. Strong cationic surfaces (CTAB) are haemolytic and cytotoxic, even when “hidden” in silica [202]. Protein corona and aggregation dictate the cellular recognition, uptake, and biodistribution of the plasmonic [218]. The shell’s integrity and porosity influence the amount of core/shell metal that escapes into the environment and its associated toxicity [219], while the multi-component “Trojan horse” effect explains how a biocompatible shell transports a more toxic core into cells [207].

Toxicity may arise from the plasmonic effects, since core–shell plasmonic nanocomposites exposed to light (for imaging, PTT, or sensing) induce photothermal heating and enhanced ROS generation. Increased local temperature during PTT destroys tumor cells, but off-target heating can also damage healthy tissues. Oxidative stress/ROS causes lipid peroxidation, DNA damage, apoptosis, and inflammation [220]. However, plasmon excitation promotes ROS production, which is helpful against cancer and bacteria but induces oxidative stress-mediated toxicity in normal cells and in the environment [221]. The cytotoxicity response is alleviated by introducing safer-by-design alterations in plasmonic core–shells.

Safer-by-design strategies [222] addressed all of those main issues to be targeted: composition, size and shape, surface charge and ligand chemistry, protein corona engineering, degradability, and persistence. For example, in composition, selected inert or relatively passive cores such as Au rather than bare Ag, well-coated iron oxides [201] or alloyed Au-Ag with a lower Ag percentage instead of a thick Ag shell, or an Au shell over Ag with non-porous intermediary layers, may reduce Ag’s access. Thin shells or alloy structures should be used to control dissolution and characterize Ag^+^ release under realistic conditions, if antimicrobial or SERS performance is required [205]. Au-only plasmonics (nanorods, nanostars) with tuned aspect ratio and coatings, as opposed to Ag, would be preferred for purely imaging/sensing uses. Also, adopting alternative nanostructures such as amorphous (silica, organosilica) or polymer (PEG, polyvinylpyrrolidone (PVP), zwitterionic) [203,208] as biocompatible shells over direct CTAB-capped surfaces and avoiding heavy-metal dopants or catalysts in the final construct will be valid options for improved biocompatibility [204]. The same technique to prevent toxicity, such as non-therapeutic use of ultra-small particles that miss critical barriers and excessively reactive shapes, may also be adopted [216]. When targeting hydrodynamic sizes for systemic applications, it should also be observed that NPs of 30–80 nm have the highest renal clearance but will cross a larger number of barriers and will interact strongly with DNA. On the other hand, very large/agglomerated particles are captured by macrophages and face increased embolic risk. Preventing sharp spikes or very high aspect ratios (if not necessary) and preparing the round or mildly anisotropic shape with a smooth silica shell minimizes membrane piercing and associated toxic effects [223]. For the design of the systems, the control of the surface charge and ligand chemistry, and for near neutral to slightly negative zeta potential in biological media (−10 to −25 mV) rather than strongly cationic surfaces, which are hemolytic and pro-inflammatory, is also a strategy to improve biocompatibility and the potential role of nanomaterials in medicine [224]. Nanotoxicology results have demonstrated that CTAB-templated silica remains highly cytotoxic because of residual CTAB, supporting the replacement of CTAB with thiolated PEG, phospholipids, or zwitterionic polymers as soon as possible in synthesis. Similarly, targeting of ligands with short, stable linkers to prevent desorption and exposure of bare metal is another potential solution [202]. Hence, favoring “stealthy” coatings (PEG, polysaccharides, proteins) instead of highly cationic ligands for systemic use could optimize the surface chemistry and biocompatibility [225]. Given that the protein corona, the NP–protein complex associated with the “true identity” of the administered NPs, has a critical role in the cell effects of NPs, intentionally engineering the corona to adsorb albumin, serum proteins, or biomimetic cell-membrane coatings, presenting a “self” interface, lowering opsonization and unspecific uptake, opens avenues in pharmaceutical science. The expected results relate to predicting and controlling the pharmacokinetics of bare or drug-loaded NPs, including systemic circulation, biodistribution, and bioavailability [218]. Therefore, using PEG with an optimized density can assist in reducing nonspecific protein adsorption in a controlled manner, without destabilizing or causing stealth-related immune issues [226].

The NPs’ degradability is also another consideration. It is desirable to design shells that dissolve to harmless species (e.g., silica into silicic acid) over biologically relevant time scales rather than highly persistent, non-degradable coatings. Silica NP-based formulations entered Phase I and Phase II trials based on the preclinical performance. The design aim is to keep the total metal dose low for “use-then-clear” constructs rather than permanent implants, unless necessary [204]. Importantly, reviews consistently emphasize that toxicity is not transferable from one formulation to another, meaning that small changes in synthesis, shell thickness, or ligands can completely change the biological profile, so each nanocomposite needs its own standardized in vitro and in vivo assessment [227].

To conclude, for Au, Ag, SiO_2_, magnetic, and other plasmonic materials, the principal toxicity triggers are dissolved ions, oxidative stress/ROS, surface charge, surfactants, size/shape, protein corona, and aggregation. For core–shell plasmonic systems, we incorporate shell integrity, porosity, and multi-component “Trojan horse” effects. Any structural architecture that makes Ag accessible to the medium (thin shell, porous silica, defects) poses a non-trivial risk of hemotoxicity and reproductive/developmental toxicity via ion release and ROS. Silica shells can be safer, but only if:

- their thickness and porosity are controlled, and
- toxic templating surfactants are removed or exchanged.

### 6.2. Clearance Pathways: Renal, Hepatic, RES-Mediated

The translation of NP-based technologies into clinical practice has been heavily stymied because of the toxic consequences that are induced by their nonspecific accumulation in healthy tissues/organs. Thus, the toxicology of plasmonic NPs involves their clearance from the plasma, mainly through renal filtration and hepatic metabolic transformations. With the generic plasmonic core (e.g., Au, Ag, Cu, CuS) and the shell (silica, polymer, iron oxide, other oxides), their renal clearance is size-dependent in relation to the glomerular filtration threshold, whose direct proportional hydrodynamic diameter (HD) must be less than 5–6 nm for adequate clearance [228]. Ultrasmall gold nanoclusters (e.g., 1–3 nm Au cores, GSH, or other small ligands) are rapidly cleared from the urine, with minimal long-term retention or overt organ toxicity, even at relatively high doses [229]. If the HD of the core–shell construct is larger than 8–10 nm, poor renal clearance is essentially assumed unless the shell is degradable into sub-6 nm units. Therefore, genuine intact core–shell renal clearance is practical only for ultrasmall designs (e.g., thin shells around sub-2–3 nm cores or constructs that disassemble to renal-clearable fragments).

For the majority of plasmonic core–shell particles utilized for imaging/therapy (between tens and hundreds of nm), the hepatic clearance process includes opsonization and hepatobiliary excretion. Opsonization is associated with hepatic Kupffer cells and splenic macrophages, and numerous AuNP assays (including bovine serum albumin (BSA)-coated and PEGylated) showed mainly accumulation in the liver and spleen but slow or incomplete clearance with detectable gold months post-dose [230]. Conversely, a relatively small fraction is shed to bile in feces in the excretion cycle, particularly for ~10–30 nm particles. For example, Au–SiO_2_ core–shell NPs in vivo bio-distribution in mice showed that they preferentially accumulated in the liver and spleen and were eventually but slowly cleared. Acute toxicity was low. Raman-active silica–Au NPs in mice have a similar profile—high liver/spleen signals, with no severe acute toxic effects, but long-term retention of the inorganic fractions [27,228,231]. For silica-coated metal NPs in general, silica shells can reduce core toxicity (e.g., molybdenum NPs that have silica coating show reduced damage to liver or spleen [232]), yet biodistribution still strongly favors RES organs, and clearance is slow [204]. The high-purity nanoassemblies, such as magneto-plasmonic core–satellites (e.g., Fe_3_O_4_@Au or Si@Au from laser ablation), demonstrate good biocompatibility, but again, accumulation is dominated by the reticuloendothelial system (RES), sometimes with eventual partial clearance [233]. In contrast, GSH-protected and zwitterionic Au nanoclusters (~2–3 nm HD) as renal-clearable ultrasmall plasmonic probes exhibit low RES accumulation, rapid renal clearance, and therefore minimal organ toxicity over more than 30 days [229].

As slow clearance is linked to liver and spleen load, long-term studies are necessary to follow persistent accumulation. For instance, the slow clearance of BSA-coated AuNPs in liver/spleen/kidney showed that their accumulation was correlated with a higher risk of late toxicity (e.g., subtle histopathological changes and altered gene expression) [230]. Moreover, although PEG-AuNPs resulted in decreased acute macrophage uptake, they continued to accumulate in a chronic manner, with tardive effects possibly due to a total Au burden exceeding the storage capacity of the cells [234]. In contrast, Gold–silica core–shell NPs and silica-coated upconversion NPs had low acute toxicity in vitro and in vivo at imaging-relevant doses yet displayed prominent liver/spleen distribution and slow clearance [27]. The bottom line is that many plasmonic core–shell particles were safe for short periods (no significant changes in alanine aminotransferase (ALT)/aspartate aminotransferase (AST), blood urea nitrogen (BUN)/creatinine, complete blood count (CBC)), although inorganic burden was accumulating. Therefore, attentive design and engineering can help to minimize these impacts. Several methods were proposed, including engineering renal clearance, controlling biodegradation, and minimizing RES uptake.

For example, designing HD smaller than 5–6 nm for a final fragment that remains after any disassembly would be feasible utilizing ultrasmall plasmonic cores (e.g., Au or CuS nanodots smaller than 3 nm) with a thin organic shell, or with a larger core–shell that cleaves into renal-clearable subunits after payload delivery (e.g., enzyme- or pH-labile linkers between satellites and core) [228]. Also possible is the use of degradable shells or linkers. In relation to the silica shell, one should consider optimizing porosity and condensation such that the NPs hydrolyze over weeks to silicic acid, which is renally excreted, or avoid highly condensed, non-erodible silica [204]. Meanwhile, biodegradable polymers such as polyesters (polylactic acid (PLA)/poly(lactic-co-glycolic acid) (PLGA)), polypeptides, or disulfide-containing polymers can be used as linkers that respond to intracellular redox conditions, capable of breaking down large constructs into smaller moieties with acceptable clearance. In such cases, bioresorbable or endogenous cores should be used whenever feasible. In high-dose or chronic applications, it is safer to use NPs capable of entering the metabolic pathways, such as iron oxide (magnetic–plasmonic) or doped CaP instead of non-degradable heavy metals [233].

In addition, even without forming “forever” particles, it is feasible to reduce RES uptake by modifying surface chemistry, shape, size, and administered dose. Using the traditional “stealth” effect, which reduces opsonization and RES uptake, improves the biokinetics of dense PEG or zwitterionic coatings [234]. However, PEG-AuNPs can still accumulate long-term. Hence, stealth combined with degradability (e.g., PEG on a cleavable shell or PEG with cleavable anchoring groups) might be the solution for improved clearance and lower toxicity [235]. Furthermore, one potential modality using the chemical surface during NP design is to avoid a strong positive charge, aiming for a near-neutral or slightly negative ζ-potential that can mitigate non-specific protein binding and membrane disruption. Because very high-aspect-ratio rods and plates have lower clearance and different macrophage uptake kinetics, quasi-spherical, compact constructs can be developed. These NPs are generally more predictable in clearance models, resulting in safer profiles [228]. After safe profiles are modelled, local or intra-tumoral delivery, plus single-shot or short-course regimens, could also modulate the kinetics and enhance clearance, thereby lowering toxicity.

In conclusion, the evidence for core–shell plasmonic NCs indicates that:

- acute safety is often fine,
- the real risk is long-term RES burden plus low-grade immunotoxic and oxidative effects.

Therefore, safer design tweaks are possible when engineering for renal clearance, controlled biodegradation, or minimizing RES uptake without creating permanent particles. Such controlled renal clearance approaches encompass engineering ultrasmall particles (HD < 5–6 nm), cores (e.g., <3 nm Au or CuS nanodots), and using degradable shells or linkers (tuned porosity and condensation), as well as bioresorbable or endogenous cores (magnetic plasmonic, doped CaP).

### 6.3. Long-Term Effects on Immunogenicity

When injected or implanted with plasmonic nanomaterials, these surfaces cooperate with:

- Innate immunity (Complement activation—C3b opsonization, Uptake by macrophages/dendritic cells, Inflammatory cytokine release -TNF-α, IL-6, IL-1β, etc) and
- Adaptive immunity (Antigen presentation, T-cell activation, Antibody production—even anti-PEG antibodies if PEGylated).

Most immunosuppression and minor chronic effects data are available for gold NPs, but some are available for Au-Ag core–shell systems. Recent immunology-focused reviews indicate that AuNPs can skew macrophage polarization, alter cytokine profiles and complement activation, and skew cytokine profiles and complement activation. The effects described have been reported depending on size, charge, and ligands [236]. Several aspects of the immunological responses to NPs’ effects were observed, and monitoring measures were used to gain insight into immunological reactivity to NPs. In particular, NPs’ persistence and organ accumulation are among the factors under investigation that may affect long-term immune adaptations. Although AuNPs are classically “immobile,” in vivo studies have demonstrated extremely slow clearance rates and long-term accumulation in RES organs (liver, spleen, kidney, and, sometimes, lung). In mice at 120 days, however, BSA-coated AuNPs were detected at high concentrations, with a ~39% reduction in liver and increased levels in spleen and kidney. This was attributed to early inflammatory and fibrotic changes in these organs [230]. In addition, AuNPs coated with PEG extended circulation but caused acute liver, kidney, and spleen damage and maintained nuclear factor-kappa B (NF-κB) activation for 90 days in reporter mice [237]. Rodent and review results revealed that AuNPs preferentially target the liver and spleen, modulating immune organs in a dose-dependent manner and conferring elevated levels of interleukin-1 beta (IL-1β), interleukin-6 (IL-6), and tumor necrosis factor (TNF-α) [238]. Consequently, any of these non-degradable core–shell plasmonic NPs is associated with prolonged residence in the liver or spleen, chronic low-grade innate immune activation, and increased fibrosis risk after high-dose or repeated dosing.

Another monitored aspect was *the effect on the innate immune activation and the complement system*. AuNPs often interact significantly with macrophages, neutrophils, and dendritic cells and have been associated with mediating or suppressing cytokine release, depending on size, coating, and dose. Pro-inflammatory and tolerogenic profiles were described [236]. Thus, cytokines such as IL-1β, IL-6, TNF-α, interleukin-8 (IL-8), and type-I interferons have been used as immunotoxicity biomarkers for nanomaterials [239]. In addition, many nano-medicines, particularly PEGylated systems (comprising molecules like drugs, proteins, or nanoparticles with one or more chains of PEG chemically attached to them), can induce complement activation-related pseudoallergy (CARPA). Anti-PEG antibodies (either already existent or induced) bind to PEGylated nanoparticles to enhance complement activation and hypersensitivity reactions [240]. When analyzing plasmonic core–shells, high-curvature protein-binding surfaces, and PEG, non-trivial CARPA risk arises, whereas shells exposing hydrophobic or cationic regions will induce complement and opsonization.

*Adaptive immunity and immunomodulation* were also assessed, as AuNPs could serve as adjuvants/antigen carriers, leading to antigen uptake, dendritic cell (DC) maturation, and antigen-specific antibody responses [241]. Notably, a 2025 comparative study reported that AuNPs significantly elevated IL-6 and TNF-α 2 h post-dose and induced strong memory T-cell activation relative to other nanoparticle types [242]. Therefore, chronically retained core–shell NPs may distort T-cell subsets (Th1/Th2/Th17, Tfh), trigger anti-ligand or anti-polymer antibodies (e.g., anti-PEG, anti-peptide), and, in extreme cases, induce immunosuppression if bone marrow or lymphoid organs are disturbed (hence T-cell-dependent antibody response (TDAR) testing).

To aggregate data on AuNPs, several studies compared silver, gold, and mixed cores/shells. For example, AgNPs are more reactive, releasing Ag^+^, forming ROS, and frequently exhibiting stronger immunotoxicity than AuNPs (inflammation, cytokine modulation, and sometimes immunosuppression at high doses) [243]. Moreover, Au-NR core/Ag-shell structures have been used as a model to examine AgNP biodistribution and its toxicity in mice. Results suggest that the toxicities resemble those of AgNPs rather than AuNPs (with liver/spleen burden and systemic effects predominantly mediated by Ag) [244]. Thus, for Au@Ag shells, the underlying immunogenicity and long-term toxicity will be more akin to silver (unless Ag is completely sealed). At the same time, Ag@Au will be relatively safer if the gold shell is defect-free. Some other results also revealed the long-term immunotoxicity of core–shell NPs. For example, in mice subjected to chronic exposure to BSA-coated AuNPs, organ levels were preserved for >120 days, spleen levels and kidney levels over time were elevated, and early inflammatory and fibrotic reactions were detected, which were more pronounced in the kidney despite the low Au content (suggesting systemic or paracrine effects). However, no catastrophic toxicity was reported, only clear months of immune activation [230,237]. Comparison with the nanomedicine immunotoxicity demonstrated the induction of IL-6/TNF-α and activation of memory T cells from the AuNPs alongside nanodiamonds. The NP biodistribution also exhibited continued organ accumulation [242]. This significantly increased both chemical and thermodynamic stability and protected the gold core from direct contact with metal and biomolecules, especially in the case of silica shells in both the Au core/silica shell and the hybrid Au–SiO_2_ NPs. Thus, silica is considered a biocompatible coating [131]. Although little long-term immuno-data can be obtained, RES accumulation and chronic exposure issues remain, but silica NP immunotoxicity is modest at low-to-moderate doses [245]. Controlled doses form the basis of the clinical translation of nanomaterials. In a Phase 0 clinical trial, high tumor accumulation of AuNPs in patients and long-term intratumoral retention were observed, with acceptable systemic safety, supporting that Au cores can themselves be tolerated in clinical practice when the drug dose is controlled [246]. By using an AuNR core/Ag shell as an Ag-toxicity model, the concentration-dependent biodistribution and toxic effects of AgNPs can be specifically detected, and it was concluded that silver dissolution dominated the toxicity profile [244]. Importantly, data suggest that a reasonable formulation with potential clinical translation is optimal, including Au, an inert shell, and controlled, well-balanced dosing. It also emphasizes the possibility that silver-bearing or highly immunogenic surface chemistries are associated with strong immune activation—particularly in the long term. Consequently, design modifications for core–shell plasmonic nanocomposites for safety must consider as many considerations as possible in order to achieve the desired response—material selection, shell and surface composition, physical parameters, biodegradability and dose strategy, and related process factors. To address the perspectives of core–shell plasmonic nanocomposites, biodegradable plasmonic systems, dynamic/transforming nanostructures, safety and immunogenic nanocomposites guidelines, and alternatives to PEG and other formulations, these are investigated [247].

In conclusion, core–shell plasmonic nanocomposites (such as Au@SiO_2_, AuNR@mSiO_2_, and PEGylated magnetic–plasmonic NPs) offer powerful optical properties with tunable immune interactions. Long-term studies show that even “safe” gold NPs can cause subtle, persistent immune and fibrotic changes, especially if they accumulate. Safer design usually means inert/degradable shells (silica, polymers), stealth coatings (PEG/albumin), appropriate size, and careful chronic in vivo immune monitoring.

### 6.4. Core–Shell Plasmonic Nanocomposites: Status of Clinical Translation and Regulatory Approval Challenges

Core–shell plasmonic nanocomposites—nanoparticles with a metallic “plasmonic” core (usually Au, Ag) and a functional shell (silica, polymers, biomolecules)—are extensively studied in biomedical applications, including imaging, diagnostics, sensing, drug delivery, and photothermal therapy. The clinical application of these materials has been sluggish, partly due to regulatory hurdles. Most plasmonic nanomaterials—especially gold-based nanoshells and nanorods—are in preclinical or early clinical testing; however, only a subset has attained full regulatory approval. One of the best-known is AuroShell^®^/Auroshell^®^ from Nanospectra Biosciences (Houston, TX, USA), as described in the US Patent 10568693 B2, US Patent 10993769 B2, US Patent 12035969 B2, and US Patent 9211419 B2). It is one of the most advanced plasmonic nanocomposites composed of silica–gold nanoshells (~150 nm) coated with PEG in pilot clinical studies [248].

Notably, AuroShell particles do not accumulate in healthy tissues, yet they are cleared from the bloodstream by RES. The particles have a 10–20 nm thick gold shell deposited on a solid silica (silicon dioxide) core and are coated with a 5000 molecular weight methoxy polyethene glycol (mPEG) chain via a thiol bond to stabilize in saline solution. After administration, the coating enhanced stability and the circulating half-life. The success in this case, Nanospectra (Aurolase^®^), is complemented by Cytimmune (Aurimune^®^), whose products are in clinical trials [249]. This is PEGylated AuNPs conjugated with recombinant human TNF-α (rhTNF), as per the report. According to reports, doses that were previously toxic when using free TNF were well tolerated in this nanoparticle formulation [250]. In addition, the study exploited the enhanced permeability and retention (EPR) effect to concentrate TNF in tumors and reduce systemic toxicity.

In contrast, clinical-trial entries and recent reviews show that combined PTT + PDT approaches using multifunctional/core–shell nanocomposites remain overwhelmingly preclinical or in early-stage translational research, with many papers describing core–shell designs that could provide both PTT and PDT in animals or in vitro but not (yet) registered as human trials on ClinicalTrials.gov. Preclinical studies are using other core–shell and hybrid NPs. For example, iron-oxide@gold core–shell nanoparticles have been studied in mouse tumor models using magnetic targeting and a NIR laser for photothermal therapy [251]. Another example is hybrid nanoplatforms combining gold with graphene (or other materials) for synergistic photothermal + photodynamic (or photochemical) activity [249].

The emerging gold nanomaterials market, with specific end-use applications, is supported by undeniable clinical and financial benefits, helping bridge the gap between academia, R&D companies, regulatory agencies, and the pharmaceutical industry.

However, there are still challenges to be addressed, such as those related to safety and toxicity, manufacturing and reproducibility, regulatory and guidance, clinical trial designs, or public or commercial banners. The long-term toxicity of metallic (especially noble-metal) nanoparticles is due to cumulative kinetics that are not fully understood [252]. Moreover, size, shape, surface coating (e.g., PEG), and shell/core design strongly influence the toxicity and biodistribution of AuNPs, which are generally considered biocompatible. Meanwhile, scalable synthesis, which requires consistency (same size, shape, shell thickness, surface chemistry), is technically challenging [170]. There is a need for quality surface engineering (e.g., PEG, targeting ligands) to ensure reliably functionalized, stable, and reproducible surfaces [253]. However, safety characterization is demanded for each part—the core, the shell, the outer coating, any possible functional cargo—which is why more detailed studies are needed than for small molecule drugs. As a result, specialized regulatory pathways and guidance are suggested to alleviate the gap between benchtop and market through routine clinical trials, toxicological testing, and regulatory categorization [248]. The element of complex regulatory maps should be avoided and replaced with specific development strategies, including the following: clear strategies for comprehensive toxicology, safety, immunogenicity, biodistribution, clearance, and metabolism studies. The findings will clarify confusion about the development strategy, stemming from the regulatory environment, and will lead us to formulate the regulated nanocomposite as a drug, biologic, device/combination product [252]. The clinical trial protocol has been approved, as it adds to previous challenges by depending on photothermal or photochemical treatments to provide even heating at the required wavelength and depth of irradiation. The dose of nanoparticles and irradiation parameters (power, duration) are important parameters. Finally, identification of the proper indication (types/location of tumor) guarantees the effectiveness and feasibility of the photothermal treatment. Otherwise, if the benefits do not outweigh the risks, the method will face public or commercial bans for clinical, technical, or legislative reasons.

The status of synergistic photothermal and photochemical (dual-mode) nanocomposites containing gold and photosensitizers, graphene, or dyes, which offer preclinical progress, is solid [249]. Dual-mode systems can potentially reduce the dose of each component, increase therapeutic efficacy, and overcome limitations such as tumor hypoxia or limited light penetration. However, despite promising in vitro/animal data, there are very few (if any) registered clinical trials specifically for core–shell plasmonic nanocomposites with both PTT + PDT activity. More advanced clinical systems (like AuroShell) focused primarily on photothermal ablation, not combined photochemistry [248]. Combining photothermal with photochemical modalities increases the regulatory complexity, as it implies effectively dealing with a multi-functional therapy (nanoparticle + light + possibly drug or photosensitizer), which may require more rigorous testing (safety, efficacy, dosimetry). Demonstrating clear clinical need and cost–benefit will attract investment. Therefore, emerging clear business or funding models need strong partnerships between academia, industry, and regulatory bodies to share the cost burden.

## 7. Challenges and Perspectives

As described, core–shell plasmonic nanocomposites offer several interesting applications in biomedical research. Despite their proven power of employing synergistic photothermal–chemical effects, the clinical use of core–shell plasmonic nanocomposites has been challenged by several systemic roadblocks.

*Design* and *Manufacturing.* Nanocomposite designs need to accurately regulate corrosion kinetics to obtain ideal pharmacokinetics and long-acting therapy. More advanced structures, such as nanostars or multicoupled core–shell–shell nanorods, offer better optical performance; however, many of these are complex morphologies that are difficult to form and are not ready for batch-to-batch manufacture. Therefore, relatively simple, tunable architectures, e.g., Ag/SiO_2_ aggregates, and robust green synthesis methods are increasingly in demand, as they provide scalable, reproducible pathways and improved regulatory properties. Increasing the therapeutic window is another major direction.

Achieving *uniform core–shell structures with precise control over core size*, *shell thickness*, *and interface properties* remains a significant challenge. Lattice mismatches in heterostructures, such as those between metal cores (e.g., Au, Ag) and semiconductor shells (e.g., TiO_2_, ZnO), can lead to defects, interdiffusion, or incomplete passivation, resulting in non-tunability of LSPR and low charge-transfer efficiency. Growth in noble-metal-based nanocrystals commonly results in irregular morphology, which can reduce the electromagnetic field enhancement of such nanocarbons, a property important for energy applications (e.g., SERS) and photothermal conversion [245].

*Biocompatibility.* Prolonged exposure to metallic NPs—especially Ag and Pd-based systems—can induce oxidative stress. To mitigate this, optimizing the core–shell architecture is critical. For instance, studies on Au@Ag nanoparticles demonstrate that precisely tuning the shell thickness (e.g., to 5 nm) can balance microbial potency with biocompatibility, maintaining 99% host cell viability while effectively killing bacteria [117]. Furthermore, the “gold shield” effect in Ag@Au hybrids has been shown to prevent rapid silver dissolution and subsequent accumulation in ferritin, thereby significantly reducing intracellular toxicity compared to bare Ag NPs [254].

*Clearence.* Balancing shelf-life with biological clearance remains a challenge. While encapsulating Ag in a Au shell (Ag@Au) prevents premature oxidation [254], hermetic sealing can block ion release. Conversely, alloying silver with platinum (AgPt) increases the oxidation activation energy, extending the antibacterial “lifetime” without halting ion release [120]. Another ongoing challenge is balancing material stability against biological removal. Even though encapsulating Ag in an Au shell (Ag@Au) does not induce premature oxidation, excess passivation can hinder therapeutic ion release. Future designs must fine-tune this “corrosion rate” for optimal pharmacokinetics.

*Integration into Clinical Platforms*. Translating these nanocomposites into wearable, injectable, or implantable devices poses hurdles in delivery and monitoring. Intravenous administration is limited by enhanced variability in the permeability and retention (EPR) effect across heterogeneous tumors, and intratumoral injections restrict broader use. Real-time dosimetry for light activation (i.e., NIR wavelengths) requires integration with imaging modalities such as photoacoustics, but equipment costs and spectral overlaps in dual systems complicate this approach [168,255,256]. Ethical and regulatory reasons, such as the requirement of the availability of long-term safety data in a variety of preclinical models, additionally contribute to hindrance in the progression toward human trials.

*Emerging Directions.* For the efficient treatment of infections in tissues (e.g., osseous and soft tissues), the second NIR (1000–1700 nm) window is also attracting interest due to its deeper tissue penetration and reduced photodamage. As a result, more research is needed to focus on the reprogramming of plasmonic activity in the NIR-II window. Au@Cu_2−x_S nanostructures demonstrated broadband absorption at 1064 nm, enabling deeper tissue penetration due to enhanced band absorption [117]. Magnetic–plasmonic hybrids, combining magnetic cores with plasmonic shells, could enable MRI-guided targeting and synergistic magnetic hyperthermia [167]. AI-guided nanomaterial design, using machine learning to predict optimal architectures from synthesis parameters, holds potential for rapid iteration and defect minimization [168,257]. Stimuli-responsive systems (pH-, enzyme-, or light-triggered for on-demand ROS/heat release) will enhance precision in drug delivery and theranostics. Multi-modal platforms integrating PDT/PTT with immunotherapy or gene editing could amplify abscopal effects, while all-organic or biodegradable core–shell variants address toxicity concerns [168,258,259,260]. Comprehensive in vivo studies, focusing on pharmacokinetics and immune modulation, alongside scalable green syntheses, will bridge the gap to personalized medicine [167,168,256,261]. Future advancements may involve integrating these systems with microfluidics or wearable devices for point-of-care diagnostics.

Specifically important *regulatory components* in the field need a boost to support further innovation. The following primary factors facilitate the move of core–shell plasmonic nanocomposites from benchtop to clinical and industrial translation:

- Strong Preclinical Efforts (more long-term toxicity/biodistribution/clearance studies and more human-tumor-mimicking in vivo models),
- Optimized Design for Translation (engineering for effective NIR windows, scaling up synthesis under Good Manufacturing Practice (GMP)-like conditions, surface adjustments for biocompatibility and targeting),
- Regulatory Strategy (early involvement with Food and Drug Administration (FDA) or European Medicines Agency (EMA), regulatory support programs),
- Clinical Trial Planning (design for safety, dose escalation, light dose, patient selection, and delivery systems),
- Business/Funding Models (collaboration among academia, industry, and regulatory bodies).

While core–shell plasmonic nanocomposites have shown remarkable promise in harnessing synergistic photothermal and photochemical effects for biomedical applications, several challenges persist. Addressing them will require interdisciplinary innovation to unlock the full potential of the field [262].

## 8. Conclusions

Core–shell plasmonic nanocomposites are innovative nanomaterials comprising a core material wrapped in a shell, with at least one component displaying plasmonic behavior. Commonly using noble metals like gold, silver, or copper, these structures offer tunable optical, electronic, and chemical properties that single-component nanoparticles cannot achieve. This review explores the connection between plasmonic fundamentals and core–shell architecture, design, coupling, tunability, biomedical applications, and legal concerns.

*Mechanisms.* Core–shell plasmonic nanocomposites are a significant advancement in material science. They combine localized surface plasmon resonances (LSPRs) with interfacial charge transfer. When optically excited, these composites enhance light absorption and scattering through electron oscillations. Their resonance characteristics can be fine-tuned based on core material and shell thickness, leading to stronger light–matter interactions. This results in improved photocatalytic activities and localized heating. The stable core–shell structure also resists environmental degradation, making these nanocomposites vital for innovative biomedical solutions.

*Architecture.* While multifunctional core–shell composites are promising for cancer therapies, their complexity can hinder mass production. For instance, photodynamic therapy (PDT) often relies on additional organic dyes, but simpler composites like Au@TiO_2_ and Au@Pd can be effective without them. This discovery encourages exploration into simpler core–shell nanoparticles for more efficient synergistic therapies, enhancing cancer treatment options.

*Design.* This review highlights advancements in core–shell plasmonic nanoparticles for synergistic photothermal and photochemical therapy. By generating heat from light-responsive cores, these nanoparticles activate normal tissue oxygen to produce cytotoxic reactive species. This dual approach broadens applications in drug delivery and cancer treatment. Careful tuning of LSPR frequency and intensity can minimize toxicity, ensure precise targeting, and support prolonged biodistribution, fulfilling diverse biomedical needs.

*Applications.* Plasmonic nanocomposites are set to revolutionize antimicrobial therapy by utilizing advanced photothermal and photochemical mechanisms to target planktonic bacteria and biofilms. Architectures like Au@TiO_2_, Au@Pd, and Ag@Au enable precise control over light absorption, heat generation, and reactive oxygen species (ROS) production, making them effective against drug-resistant pathogens. By combining localized hyperthermia with oxidative stress, these nanomaterials disrupt microbial membranes and penetrate biofilms. Future research should focus on improving biosafety, scalability, and in vivo delivery to enhance their clinical potential in combating multidrug-resistant bacteria.

The *combination of photothermal ablation and ROS-mediated apoptosis*, especially with ligand-based targeting, has the potential to enhance cancer treatment. Multifunctional nanoplatforms that offer real-time imaging, targeted delivery, and dual-mode cytotoxicity are emerging as effective solutions to the limitations of traditional therapies. Although challenges remain for clinical adoption, advancements in core–shell systems show promise for precision oncology. Core–shell plasmonic nanocomposites are transforming biosensing and bioimaging through enhanced optical properties. Their ability to support SERS, fluorescence, and photoacoustic imaging positions them as promising candidates for next-generation diagnostic tools in precision medicine.

The *synergistic photothermal and photochemical core–shell nanocomposite* offers significant benefits for wound healing and tissue regeneration. It can damage bacteria and biofilms while enhancing healing processes and drug penetration. Additionally, it generates ROS to kill bacteria and modulate inflammation. Topical therapies using hydrogel nanoparticles maintain a moist healing environment and support tissue growth. However, challenges such as biocompatibility and inflammation management must be addressed for successful clinical application.

*Toxicity.* In plasmonic materials like gold (Au), silver (Ag), and silica (SiO_2_), toxicity is influenced by factors such as dissolved ions, oxidative stress, reactive oxygen species (ROS), surface charge, surfactants, size and shape, protein corona, and aggregation. For core–shell systems, additional risks include shell integrity and porosity. Designs exposing silver to the medium—such as thin shells or porous silica—pose significant risks of hemotoxicity and developmental toxicity due to ion release and ROS. Silica shells can improve safety if shell thickness and porosity are controlled and harmful surfactants are removed. Studies show that while acute safety may be acceptable, long-term accumulation can lead to chronic immunotoxic and oxidative effects. Safer design modifications include engineering ultrasmall particles for enhanced renal clearance, using degradable shells, and incorporating bioresorbable cores. Core–shell nanocomposites, like Au@SiO_2_ and PEGylated magnetic-plasmonic nanoparticles, offer excellent optical properties and adaptable immune interactions. Even “safe” gold nanoparticles can cause persistent immune responses and fibrosis if they accumulate, so safer designs should include inert shells and stealth coatings, along with careful immune monitoring.

*Legal.* To advance the field, several areas need attention: (1) robust preclinical studies for long-term toxicity and biodistribution; (2) optimized designs for better translation to clinical use; (3) regulatory strategies with early engagement from agencies like the FDA; (4) thorough clinical trial planning; and (5) effective business models fostering partnerships between academia, industry, and regulatory bodies. Clear clinical needs and cost–benefit evidence will attract investment.

In conclusion, as we address challenges in synthesis control and biocompatibility, opportunities for deeper research, NIR-II responsive hybrids, and AI-optimized designs arise. Plasmonic nanocomposites represent a next-generation platform with precise control over light absorption, heat generation, and ROS production, offering significant therapeutic potential.

## Figures and Tables

**Table 1:** Main historical milestones in the development of core–shell plasmonic mamocomposite.

Year	Material/Concept	What Changed	Biomedical Impact	Ref.
1938	TiO_2_ dye photobleaching (Goodeve–Kitchener)	Early photocatalysis on TiO_2_	Established oxide-surface photo-redox baseline	[9]
1972	Honda–Fujishima water splitting	Semiconductor band-edge redox	Anchored TiO_2_ as safe photocatalyst candidate	[10]
1968–1974	Kretschmann/SPR; SERS	Excitable surface plasmons; field enhancement	Biosensing	[11]
2003–2008	Au nanoshells/AuNR PTT	Tunable NIR absorption; strong PCE	First robust in-tissue photothermal ablations; clinical pilot	[12]
2018	TiO_2_-capped AuNRs	Plasmon-boosted ^1^O_2_ and heat generation (synergy)	Improved performance at lower dose/temperature	[13]
2018	AuNR–TiO_2_ nanoclusters	Broad spectrum 500–1000 nm ROS and PTT (synergy)	Clear in vitro PDT and PTT synergy	[14]
2019	Au@TiO_2_ (zwitterionic-gated)	Single-wavelength PTT + PDT (synergy)	In vitro/in vivo combined therapy; drug-loading	[15]
2022	UCNP@AgBiS_2_	UC-assisted PDT + strong PTT (synergy)	In vivo ablation under NIR windows	[16]
2021–2024	Au/MoS_2_, MOF shells	NIR catalysis, PS confinement, O_2_ management	Higher ROS with matched heating; toward single-beam treatment	[17]

**Table 2:** Examples of core–shell architectures used in biomedical applications.

Core–Shell	Material	Size	Irradiation	Application	Reference
Metal-SiO_2_	Ag@SiO_2_	NP: 20–40 nm shell: 2–3 nm	LED, 410 nm, 14–86 J cm^−2^, 16 h	Cancer therapy	[47]
	Ag nanocubes @SiO_2_-RB	core: 44 nm shell: 10 nm	LED, 520 nm, 20 W, 2–4 h	Antibacterial	[50]
	Au@SiO_2_-MB	core: 40 nm shell: 40 nm	LED, 590 nm, 5 mW cm^−2^, 20 min	Cancer therapy	[48]
	Au nanorods @SiO_2_-TCPP	core: 53/13.5 nm shell: 66 nm	Laser, 660 nm (0.5 W cm^−2^) and Laser, 808 nm (2 W cm^−2^), 5 min	Cancer therapy	[49]
	Au nanorods@SiO_2_–TPP (TPP encapsulated into conjugated polymer particles)	core: 50/12.5 nm shell: 9–12 nm	Laser, 800 nm, 0.38 mJ cm^−2^, 8 min	Cancer therapy	[64]
	Au nanorods@SiO_2_-IR795	core: 57/17 nm shell: 12 nm	Laser, 808 nm, 1 W cm^−2^, 5 min	Cancer therapy	[51]
	Au@SiO_2_@Au (functionalized with hyaluronic acid)	NP: 37.5 nm	Laser, 808 nm, 1.5 W cm^−2^, 10 min	Cancer therapy	[65]
	SiO_2_@Au	core: 145 nm shell: 20 nm	Laser, 810 nm, 2.5 W cm^−2^, 10 min	Antibacterial	[29]
	SiO_2_@Au@SiO_2_	NP:480–510 nm	Laser, 660 nm, 2 W cm^−2^, 2 min	Cancer therapy	[22]
	SiO_2_@AuAg/PDA	NP: 330 nm shell: 25 nm	Laser, 808 nm, 2.5 W cm^−2^, 6 min	Antibacterial	[44]
Metal-semiconductor	Au@TiO_2_ (with DOX loading and a coating of a pH-sensitive polymer)	NP: 108 nm shell: 32 nm	Laser, 635 nm, 2 W cm^−2^, 5 min	Cancer therapy	[15]
	Au@MnO_2_	NP: 110 nm shell: 35 nm	Laser, 808 nm, 0.25 W, 4 min	Sensing Cancer therapy	[52]
	Au-Ag@MnO_2_	NP: 59–174 nm core: 49 nm	Laser, 785 nm, 0.5 W	Sensing	[66]
	PEG-Au@CuS	NP: 70 nm core: 35 nm	Laser, 785 nm, 84.5 mW; Laser, 808 nm, 1 W cm^−2^, 10 min	Sensing Cancer therapy	[45]
	Au nanorods @SiO_2_@CuS (with a polycationic cloak)	NP: 55/80 nm core: 10/40 nm	Laser, 1064 nm, 0.5 W cm^−2^, 5 min	Cancer therapy	[67]
	Au@CuS@CuO_2_	NP: 40 nm	Laser, 785 nm, 10 mW	Sensing	[43]
	Au@CuS@CuO_2_	NP: 120 nm	Laser, 808 nm, 1 W cm^−2^, 5 min	Cancer therapy	[42]
Metal-Fe_3_O_4_	Ag@Fe_3_O_4_	NP: 250 nm shell: 80 nm	Laser, 808 nm, 2 W cm^−2^, 10 min	Cancer therapy	[68]
	Fe_3_O_4_ @Au	-	Laser, 808 nm, 1 W cm^−2^, 10 min	Cancer therapy	[53]
	Fe_3_O_4_ @Au (with PEI modification and COOH-PEG-FA grafting)	NP: 150 nm	Laser, 808 nm, 1 W cm^−2^, 5 min	Cancer therapy	[69]
	Fe_3_O_4_ @Au (surface modified with aptamer)	NP: 80 nm shell: 70 nm	Laser, 808 nm, 1.5 W cm^−2^, 5 min	Sensing Antibacterial	[70]
Metal @ Metal	Au@Ag@PDA	NP: 40 nm Au core: 18 nm	Laser, 785 nm, 0.5 mW	Sensing	[71]
	Au@Ag nanoislands	nanoislands height: 59 nm	Simulated sunlight, 632 nm, 0.3 W cm^−2^, 10 min	Antibacterial Sensing	[46]
	Au nanorods@Pd	core: 76 nm shell: 5 nm	Laser, 1064 nm, 1 W cm^−2^, 5 min	Cancer therapy	[72]
	Ag@Au	NP: 70–140 nm core: 15–22 nm	Laser, 671 nm, 3 mW	Sensing	[73]
Metal @ organic macromolecules	graphene oxide-Au@PANI	NP: 120 nm	Laser, 808 nm, 2.5 W cm^−2^, 10 min; 785 nm 514 nm	Cancer therapy Sensing	[74]
	Au nanobipyramids @PDA	NP: 58/109 nm	Laser, 808 nm, 0.5 W cm^−2^, 10 min	Cancer therapy	[75]
	Au@Cu_3_(BTC)_2_	NP: 43 nm	Laser, 532 nm, 30 mW; Laser, 808 nm, 2 W cm^−2^, 10 min	Sensing Cancer therapy	[76]
	Au nanorods@ZIF-8	core aspect ratio: 3.9 shell: 25 nm	Laser, 808 nm, 1 W cm^−2^, 10 min	Cancer therapy	[24]
	Au@ZrTCPP (Encapsulated with gambogic acid + coated with PEGylated liposome)	NP: 88 nm core: 50 nm	Laser, 980 nm, 0.5 W cm^−2^, 10 min	Cancer therapy	[77]

**Table 3:** Main examples of core–shell and hybrid systems used in antimicrobial therapy.

Nanocomposite System	Structure Type	Synergistic Mechanism	Irradiation (Range)	Target Microorganisms	Ref.
Au@Ag	Core–Shell	Gold shell hermetically seals Ag core to prevent rapid oxidation while allowing PTT.	NIR	*Staphylococcus aureus* + other general bacteria	[113]
Au@Ag	Core–Shell	PTT downregulates *fabF* gene (genetic modulation); reduces membrane fluidity; Ag^+^ release.	NIR (808 nm)	*Enterococcus faecalis*	[114]
Au@Ag (Green)	Core–Shell (Green)	Multi-faceted mechanism (Ag^+^ + ROS) with linear concentration-inhibition correlation.	Contact/Intrinsic	*E. coli*, *B. subtilis*	[115]
Au/Ag	Decentralized Core–Shell	Au core supports Ag shell; Ag shell releases Ag^+^, generates ROS, and disrupts DNA via electrostatic binding.	Intrinsic	*Pseudomonas aeruginosa* (Max inhibition), *Shigella flexneri*, *MRSA*.	[116]
Au@Ag (Optimized)	Core–Shell (Variable)	Thinner shells (5 nm) release more Ag^+^ than thicker shells due to interface.	Intrinsic	*E. coli*, *S. aureus*	[117]
AuAgNS (Au Nanovesicles)	Hollow Nanoshells	Hyperthermia + sustained Ag^+^ ion release.	NIR (1.0 W/cm^2^)	MRSA, ESBL *Escherichia coli*, *Staphylococcus aureus*	[118]
Ag@Au-Chitosan-Ciprofloxacin	Core–shell NPs loaded with Ciprofloxacin	Synergy between the Ag@Au core–shell structure, the chitosan stabilizer, and the loaded antibiotic.	-	*E. coli*, *S. aureus*, *B. subtilis*, *P. mirabilis*	[119]
Au–Ag–Au	Core–shell–shell (Nanorods)	Low-power heat (44 °C) melts outer Au shell to expose bactericidal Ag.	NIR (50 mW/cm^2^)	*Escherichia coli* O157:H7	[103]
Au@AgPt	Nanorods core with alloy nanodots shell	Pt increases activation energy for Ag oxidation; extends antibacterial lifetime + PTT.	NIR	*E. coli*, *S. aureus*	[120]
AgPd Nanodarts	Asymmetric (Dart-like)	Record 86.7% PTT efficiency + POD-like activity.	NIR-II (1064 nm)	*Staphylococcus aureus*	[107]
Ag@ZnO	Core–Shell	Induces host antimicrobial peptides (*hBD2*, *RNase7*); enhances lysosomal degradation.	Bio-interface	Intracellular/extracellular *Staphylococcus aureus*	[109]
Ag@AgCl	Core–Shell (Cubes)	RCS generates unique chlorine radicals via photo-excited holes.	Visible (532 nm)	MRSA, *Escherichia coli*	[121]
Fe@ZnO	Core–Shell	Narrowed band gap (2.81 eV) for broad-spectrum activation.	Sunlight/UV	Degradation of antibiotic pollutants	[122]
Cu_2-x_S@MSS	Core–Shell (Silica)	PTT + Fenton-like Cu_2_ release + Thermally triggered antibiotic desorption.	NIR (808 nm)	Gram-positive like *Staphylococcus aureus*	[106]
MXene-Au@Ag	2D/0D Hybrid	Physical slicing of membranes by MXene edges + Chemical toxicity (Ag^+^)	Contact/Light	*Staphylococcus aureus*, *Escherichia coli*	[112]

**Table 4:** Examples of core–shell nanocomposites used in dual-mode imaging and cancer therapy (theranostics).

Material System	Plasmonic Effect	Photodynamic/Photothermal Synergism	Core–Shell/Structural Design	Noble/Non-Noble Composition	Theranostics	Key Findings	Ref.
Fe_3_O_4_@Au	LSPR NIR absorption (~670 nm) light–heat conversion	Photothermal + mild ROS under NIR; selective thermal ablation	Fe_3_O_4_ magnetic core coated with Au shell; S6 aptamer-targeting	Au (plasmonic, stable) + Fe_3_O_4_ (magnetic)	Magnetic targeting + plasmonic photothermal destruction	Combines magnetic isolation, fluorescence detection, and selective thermal ablation	[130]
Au nanorod mesoporous SiO_2_, PdTPP	Strong TPL and LSPR; PRET	Two-photon-activated PDT via Au → PdTPP → O_2_ pathway; ROS minimal heating	Core–shell: AuNR core + mesoporous SiO_2_ shell (photosensitizer loading)	Au (plasmonic) + SiO_2_ (porous stabilizer)	Two-photon luminescence (TPL) + singlet-O_2_-mediated	Enables deep-tissue two-photon imaging and plasmonic PDT with excellent photostability	[131]
Mn@ Polymer@Au @TiO_2_ @DOX (multi-shell)	LSPR in Au core enhances charge transfer to TiO_2_, optical response to NIR	Au induces heat (PTT) + TiO_2_ produces ROS (PDT) + DOX for chemo	Multilayer core–shell: Au@TiO_2_ core + zwitterionic polymer + DOX + Mn^2+^ for MRI	Au (plasmonic) + TiO_2_ (semiconductor) + Mn (magnetic)	MRI contrast imaging + PTT/PDT/chemo therapy (multimodal)	Enables MRI-guided synergistic phototherapy and drug delivery; pH-sensitive polymer gating	[15]
Au/Ag bimetallic porous hybrids; Au@Ag, Au@MOF	Hybridized LSPR; Au–Ag absorption; porous structure light scattering	Combined PTT and imaging-guided therapy; synergy with chemotherapy and radiotherapy	Porous and hollow nanostructures (MOFs, silica, carbons) with plasmonic metal doping	Au/Ag (plasmonic) + MOF, silica, carbon	Optical/Photoacoustic/CT imaging + PTT (Imaging-guided theranostics)	Focus on plasmonic porous platforms for imaging-guided photothermal ablation and drug delivery	[129]

**Table 5:** Cross-study insights.

Category	Noticed Trend (2012…)	Implication for Theranostics
Plasmonic function	Evolved from pure heating [130] → plasmonic energy transfer [131] → charge-separation enhancement [15] → imaging-guided tuning [129]	Integration of photonics, catalysis, and imaging at nanoscale precision
Therapeutic mechanism	Thermal ablation → ROS-driven apoptosis → multi-modal synergy (PTT + PDT + Chemo)	Multi-functional “smart” therapy systems replacing single-modality approaches
Core–shell engineering	Fe_3_O_4_@Au → Au@SiO_2_ → Au@TiO_2_@Polymer → Au@Ag@MOF	Increasing structural complexity and functional layering for enhanced stability and multimodality

## Data Availability

Not applicable.

## Abbreviations

The following abbreviations are used in this manuscript:

ALT	Alanine Aminotransferase
AM	Acrylamide
AST	Aspartate Aminotransferase
BSA	Bovine Serum Albumin
BUN	Blood Urea Nitrogen
CARPA	Complement Activation-Related Pseudoallergy
CBC	Complete Blood Count
CDT	Chemodynamic Therapy
CHX	Chlorhexidine
cRGD	Cyclic Arginine–Glycine–Aspartic acid
CTAB	Cetyltrimethylammonium Bromide
DC	Dendritic Cell
DNA	Deoxyribonucleic Acid
DOX	Doxorubicin
EMA	European Medicines Agency
EPR	Enhanced Permeability and Retention
EPS	Extracellular Polymeric Substances
FA	Folic Acid
FDA	Food and Drug Administration
GA	Gambogic Acid
GMP	Good Manufacturing Practice
GT	Gene Therapy
FHMP	Fluorescent Hexametaphosphate
GSH	Glutathione
HD	Hydrodynamic Diameter
IL-1β	Interleukin-1 beta
IL-6	Interleukin-6
IL-8	Interleukin-8
IOC	Iron Oxide Cluster
LSPR	Localized Surface Plasmon Resonance
mAbs	Monoclonal Antibodies
MOA	Mechanisms of action
MOF	Metal–Organic Frameworks
mPEG	Methoxy Polyethene Glycol
MRI	Magnetic Resonance Imaging
MIC	Minimum Inhibitory Concentration
MIP	Molecularly Imprinted Polymer
MUA	11-Mercaptoundecanoic Acid
NIPAM	N-isopropylacrylamide
NIR	Near-Infrared
NR	Nanorods
NIR-II	Second Near-Infrared
NPs	Nanoparticles
NS	Nanosphere
NF-κB	Nuclear Factor-kappa B
PANI	Polyaniline
PBS	Phosphate-Buffered Saline
PCE	Photothermal Conversion Efficiency
PDA	Polydopamine
PDA-HA	Polydopamine–Hyaluronic Acid
PDT	Photodynamic Therapy
PEF	Plasmon-Enhanced Fluorescence
PEG	Polyethylene Glycol
PEI	Polyethyleneimine
PIRET	Plasmon-Induced Resonance Energy Transfer
PLA	Polylactic Acid
PLAL	Pulse Laser Ablation in Liquid
PLGA	Poly(lactic-co-glycolic acid)
POD	Peroxidase
PTT	Photothermal Therapy
PVP	Polyvinylpyrrolidone
RB	Rose Bengal
rBSA	Reduced Albumin from Bovine Serum
RCS	Reactive Chlorine Species
RES	Reticuloendothelial System
rhTNF	Recombinant Human Tumor Necrosis Factor
ROS	Reactive Oxygen Species
SERS	Surface-Enhanced Raman Scattering
SPION	Superparamagnetic Iron Oxide Nanoparticle
SPR	Surface Plasmon Resonance
TEOS	Tetraethyl Orthosilicate
TNF-α	Tumor Necrosis Factor
UCNP	Upconverting Nanoparticles
ZrTCPP	Zirconium Tetrakis(4-carboxyphenyl)porphyrin
